# Oral ferroportin inhibitor VIT‐2763: First‐in‐human, phase 1 study in healthy volunteers

**DOI:** 10.1002/ajh.25670

**Published:** 2019-11-19

**Authors:** Frank Richard, Jan Jaap van Lier, Bernard Roubert, Teba Haboubi, Udo‐Michael Göhring, Franz Dürrenberger

**Affiliations:** ^1^ Research and Development, Vifor Pharma Group Glattbrugg Switzerland; ^2^ Early Development Services, PRA Health Sciences Groningen Netherlands; ^3^ Chemical and Preclinical Research and Development, Vifor (International) AG St. Gallen Switzerland

## Abstract

Restriction of iron availability by ferroportin inhibition is a novel approach to treating non‐transfusion‐dependent thalassemia (β‐thalassemia intermedia). This first‐in‐human, Phase I study (https://www.clinicaltrialsregister.eu; EudraCT no. 2017‐003395‐31) assessed the safety, tolerability, pharmacokinetics and pharmacodynamics of single‐ and multiple‐ascending doses (SAD and MAD) of the oral ferroportin inhibitor, VIT‐2763, in healthy volunteers. Participants received VIT‐2763 5/15/60/120/240 mg or placebo in the SAD phase and VIT‐2763 60/120 mg once daily, VIT‐2763 60/120 mg twice daily, or placebo for 7 days in the MAD phase. Seventy‐two participants completed treatment. VIT‐2763 was well tolerated and demonstrated a similar safety profile to the placebo. There were no serious or severe adverse events, or discontinuations due to adverse events. VIT‐2763 absorption was relatively fast, with detectable levels 15 to 30 minutes post‐dose. Following multiple dosing there was no apparent change in absorption and accumulation was minimal. Mean elimination half‐life was 1.9 to 5.3 hours following single dosing, and 2.1 to 3.8 hours on Day 1 and 2.6 to 5.3 hours on Day 7, following repeated dosing. There was a temporary decrease in mean serum iron levels with VIT‐2763 single doses ≥60 mg and all multiple doses; mean calculated transferrin saturation (only assessed following multiple dosing) also temporarily decreased. A shift in mean serum hepcidin peaks followed administration of all iron‐lowering doses of VIT‐2763. This effect was less pronounced after 7 days of multiple dosing (aside from with 120 mg once daily). These results support the initiation of clinical studies in patients with non‐transfusion‐dependent thalassemia and documented iron overload due to ineffective erythropoiesis.

## 1 INTRODUCTION

Iron is essential for multiple cellular processes, with large amounts being used in the production of hemoglobin during erythropoiesis. Thus, erythropoietic demand for iron is a primary regulator of iron absorption.^1^, ^2^, ^3^ Ineffective erythropoiesis can lead to excessive iron absorption in certain inherited hemoglobin disorders, such as thalassemia and other conditions associated with variations in hemoglobin structure. The two main forms of thalassemia, α‐ and β‐thalassemia, are caused by defects in α‐ and β‐hemoglobin chain synthesis, respectively.^4^ In β‐thalassemia, partial or complete loss of β‐globin synthesis, due to mutations in the β‐globin gene, results in a relative excess of α‐chains and the formation of α‐globin‐heme complexes on red blood cell (RBC) membranes.^1^, ^5^ The severity of symptoms, which are generally related to anemia and/or iron overload, depends on the extent to which β‐globin production is diminished and the degree of α‐ and β‐chain imbalance.^4^, ^5^

Phenotypes of thalassemia and their severity can be characterized according to dependence on blood transfusions. Patients are conventionally described as having transfusion‐dependent thalassemia (TDT; β‐thalassemia major) when they have a life‐long requirement for regular transfusions, which results in iron overload.^6^, ^7^ Chronic transfusions are not essential for those with non‐transfusion‐dependent thalassemia (NTDT; β‐thalassemia intermedia) to survive, but for limited periods of time, infrequent or sometimes repeated transfusions might be necessary.^6^, ^8^, ^9^ In NTDT, ineffective erythropoiesis leads to chronic hemolytic anemia and subsequent hypoxia. This drives compensatory elevated levels of erythropoiesis and suppressed synthesis of the iron‐regulatory hormone, hepcidin. Consequently, intestinal iron absorption is increased, recycled iron from the reticuloendothelial system is released, macrophage iron is depleted, and portal and hepatocyte iron loading is increased.^1^, ^5^, ^10^, ^11^, ^12^, ^13^ Subsequently, the amount of released iron exceeds the binding capacity of transferrin and non‐transferrin‐bound iron (NTBI) is formed in the circulation.^14^, ^15^ Due to its ability to generate reactive‐oxygen species, accumulation of unbound iron in tissues can be toxic, resulting in tissue injury (including trophic skin changes and leg ulcers), organ dysfunction (including liver disease, heart failure and endocrinopathies), and death.^1^, ^7^, ^15^, ^16^

The clinical management of β‐thalassemia generally involves blood transfusions, iron chelation, and treatments to alleviate symptoms of iron overload.^17^, ^18^ Potentially curative treatments include bone marrow transplant in selected patients,^17^, ^18^ and gene therapies that aim to partially, if not fully, correct the disorder.^19^, ^20^, ^21^, ^22^, ^23^ The gene therapy, lentiglobin, which acts via the transplantation of lentiviral‐transduced hematopoietic stem cells with increased production of β‐globin,^21^, ^23^, ^24^ was approved for the treatment of TDT in the EU in June 24, 2019. Other emerging therapies are based on animal model evidence that restriction of iron to the erythron can improve ineffective erythropoiesis by decreasing the formation of RBC membrane‐associated α‐globin aggregates, and subsequent reactive‐oxygen species‐mediated oxidative stress and apoptosis.^25^, ^26^, ^27^ One such therapy, VIT‐2763, is a novel, small‐molecule oral inhibitor of ferroportin. Ferroportin is the only known iron transporter in mammals and is mainly expressed in tissues of iron absorption (duodenum), recycling and storage (liver, spleen and mononuclear phagocyte system), mediating the transfer of iron into the blood.^3^, ^28^, ^29^, ^30^, ^31^ In vitro, VIT‐2763 inhibited cellular iron efflux with a potency comparable to that of hepcidin, the natural inhibitor of ferroportin, and in preclinical studies, VIT‐2763 demonstrated good oral bioavailability.^32^ In 14‐day toxicity studies in Wistar rats, the “no observed adverse effect level” was >600 mg/kg; in longer‐term studies, no dose‐limiting toxicity was observed. Dose‐limiting effects in rodents were related to the pharmacology of VIT‐2763 (restricted iron uptake) and resulting iron deficiency anemia and subsequent secondary effects thereof.^32^ In a mouse model of NTDT, VIT‐2763 decreased serum iron levels, ameliorated anemia and improved ineffective erythropoiesis.^32^ Thus, limiting iron availability for erythropoiesis via ferroportin inhibition is a potential approach to treating NTDT.^33^

The aim of this first‐in‐human, Phase I study (EudraCT no. 2017‐003395‐31) was to assess the safety and tolerability, and to determine the pharmacokinetics (PK) and pharmacodynamics (PD), of single‐ and multiple‐ascending oral doses of VIT‐2763 in healthy volunteers.

## 2 METHODS

### 2.1 Participants

Healthy male or female volunteers aged between 18 and 65 years were eligible. Exclusion criteria comprised any history of iron storage diseases; history or clinical findings of iron utilization disorders; known hemoglobinopathy; use of intravenous iron therapy, erythropoietin stimulating agent therapy and/or blood transfusions in the previous 3 months, and/or excessive oral iron or oral iron‐containing products in the previous 4 weeks. A complete list of inclusion and exclusion criteria is provided in Table S1.

### 2.2 Study design

This Phase 1 study was a randomized, double‐blind, placebo‐controlled, parallel‐group, dose‐escalation study, and comprised a single‐ascending dose (SAD) phase and a multiple‐ascending dose (MAD) phase. The study was conducted at a PRA Health Sciences Early Development Services study site in Groningen, Netherlands, according to the principles of the Declaration of Helsinki. The study was also conducted according to the International Council for Harmonization of Technical Requirements for Registration of Pharmaceuticals for Human Use (ICH) Guidelines, following approval from the appropriate Independent Ethics Committee. All participants provided written informed consent.

Participants were admitted on Day −1 and received VIT‐2763 or placebo the following day (Day 1 in the SAD phase) or for the next 7 days (Day 1 to Day 7 in the MAD phase). Participants remained hospitalized for assessments up to and including Day 4 or Day 10 in the SAD and MAD phases, respectively, returning for a final study assessment 3 days later. Participants were assigned a randomization number prior to dosing, according to a code generated by the Biostatistics Department of PRA Health Sciences. In the SAD phase, sequential, ascending cohorts received single oral doses of VIT‐2763 (5, 15, 60, 120 or 240 mg) or matching placebo. Sentinel dosing was used, whereby the first two participants in each SAD cohort were randomized to receive either VIT‐2763 or placebo. Dosing of the remaining participants at the same dose level occurred at least 48 hours later, following a safety evaluation of the sentinel participants. Escalation to the next dose level occurred following data review by the Safety Review Committee. In the MAD phase, sequential, ascending cohorts received multiple oral doses of VIT‐2763 (60 or 120 mg, once daily [QD] or twice daily [BID]) or matching placebo for 7 days. Dosing in the MAD phase followed an assessment of data from the first four cohorts of the SAD phase. Subsequent multiple‐dose cohorts were initiated once data from the preceding SAD cohort had been evaluated, along with cumulative data from the preceding multiple‐dose cohorts. Starting and maximum doses, which were based on preclinical toxicology studies and physiologically based PK/PD modeling and simulation, were in accordance with European Medicines Agency (EMA) guidelines.^34^ If a predefined safety threshold was reached (C_max_ ≥3805 ng/mL; equivalent to the no observed effect level in dogs) dosing at that level or higher would not continue.

### 2.3 Endpoints and assessments

Primary endpoints were safety related and comprised the incidence of adverse events (AEs) and serious AEs, which were coded according to Medical Dictionary for Regulatory Activities code (version 21.0), changes from baseline in vital signs, clinical laboratory tests (hematology, biochemistry, coagulation, urinalysis), twelve‐lead electrocardiogram (ECG), cardiac telemetry, and physical examinations. The severity of AEs and relationship of AE to study drug were also assessed. Secondary endpoints were VIT‐2763 PK and PD. A complete list of endpoints is included in Table S2 and a summary of all assessments and related time points is included in Figure S1.

### 2.4 Statistical analysis

As this was a first‐in‐human study of VIT‐2763, no formal sample‐size calculation was performed. The PK and PD analyses were performed on the full analysis set (FAS), comprising all participants randomized to treatment, who received ≥1 complete dose and had ≥1 post‐baseline PK and PD assessment. The PK and PD analyses were repeated using the per‐protocol set, comprising all participants who fulfilled FAS criteria and had no major protocol violations. The safety set comprised all randomized participants who had received ≥1 dose of study medication. Safety, PK and PD statistical analyses were descriptive. Continuous variables were presented as mean, SD, SE or median, range and 95% confidence interval, as appropriate; categorical and ordinal variables were presented as frequencies and percentages. Results are presented for each VIT‐2763 dose cohort; data for participants receiving placebo were pooled across cohorts. All statistical analyses were performed using SAS Version 9.3 or higher and with WinNonlin Version 6.3 or higher. There was no imputation of missing values, except missing start and end times of AEs, and PK concentrations below lower limit of quantification (LLOQ).

### 2.5 Data‐sharing statement

The key aspects of the study protocol and fully anonymized data were submitted to the EudraCT clinical trial registry at https://www.clinicaltrialsregister.eu at the end of October 2019. Detailed inclusion criteria, study endpoints, the most common AEs and all reported AEs are included in this publication.

## 3 RESULTS

### 3.1 Participant disposition and baseline characteristics

A total of 72 participants were randomized and completed treatment. The number of participants assigned to each dose level in the SAD and MAD cohorts is shown in Figure S2. Demographic variables and baseline characteristics of the treatment cohorts in the SAD and MAD phases are presented in Table 1. A higher proportion of participants were female than male (66% and 71% of participants in the overall SAD and MAD cohorts, respectively), and the treatment cohorts were slightly imbalanced in terms of proportions of female to male participants. Median age ranged from 21.5 to 29.0 years in SAD cohorts, and from 22.5 to 33.5 years in the MAD cohorts and mean body mass index (BMI) ranged from 21.9 to 25.3 kg/m^2^ in the SAD cohorts, and from 21.8 to 24.6 kg/m^2^ in MAD cohorts. Despite differences in the proportions of female to male participants, mean baseline levels of serum iron, calculated transferrin saturation, transferrin and hemoglobin were generally comparable across treatment cohorts in both the SAD and MAD phases; during the MAD phase, median (min, max) values for hemoglobin were similar between treatment cohorts.

**Table 1:** Baseline demographics for single‐ascending dose cohorts (A) and multiple‐ascending dose cohorts (B) (safety population)

A.							
Variable	VIT‐2763 5 mg N = 6	VIT‐2763 15 mg N = 5	VIT‐2763 60 mg N = 6	VIT‐2763 120 mg N = 6	VIT‐2763 240 mg N = 6	Pooled placebo N = 9	Total N = 38
Age, years, median (min, max)	27.5 (21, 65)	24.0 (19, 63)	22.5 (18, 26)	29.0 (21, 52)	21.5 (19, 23)	23.0 (19, 56)	23.0 (18, 65)
Female sex, n (%)	2 (33)	2 (40)	4 (67)	5 (83)	4 (67)	8 (89)	25 (66)
White race, n (%)	5 (83)	5 (100)	5 (83)	6 (100)	6 (100)	6 (67)	33 (87)
Weight, kg, median (min, max)	78.6 (68.4, 93.9)	73.7 (62.2, 83.1)	68.4 (55.5, 75.0)	72.8 (60.6, 78.3)	67.4 (56.7, 82.8)	76.5 (56.5, 84.4)	72.3 (55.5, 93.9)
BMI, kg/m^2^, median (min, max)	25.5 (21.7, 28.7)	23.6 (20.1, 27.8)	21.3 (18.8, 26.4)	23.7 (21.1, 26.2)	21.8 (21.3, 23.5)	23.9 (20.9, 29.0)	23.3 (18.8, 29.0)
Serum ferritin, ng/mL, median (min, max)	89.0 (32, 198)	121.0 (43, 165)	34.5 (17, 124)	53.0 (20, 170)	68.0 (29, 132)	41.0 (15, 200)	56.0 (15, 200)
TSAT, %, median (min, max)	26.3 (15.7, 43.2)	25.7 (18.1, 40.5)	33.1 (19.3, 44.5)	28.6 (24.4, 43.0)	33.0 (13.4, 48.5)	32.0 (19.9, 49.1)	28.6 (13.4, 49.1)
Serum iron μg/dL, median (min, max)	94.5 (63.7, 124.1)	82.2 (61.5, 111.2)	92.5 (44.7, 185.6)	101.2 (69.3, 157.1)	99.8 (47.0, 128.0)	76.6 (44.7, 181.7)	91.1 (44.7, 185.6)
Serum transferrin mg/dL, median (min, max)	291.0 (237, 321)	270.0 (170, 322)	276.0 (243, 311)	252.5 (200, 300)	281.0 (234, 321)	286.0 (235, 482)	276.0 (170, 482)
Hemoglobin, g/dL, median (min, max)	14.7 (14.0, 15.6)	14.5 (13.7, 16.7)	14.2 (11.8, 14.7)	14.8 (13.2, 15.1)	14.0 (13.2, 14.8)	13.5 (13.4, 14.8)	14.2 (11.8, 16.7)
Hepcidin, ng/mL, median (min, max)	17.7 (12.8, 46.9)	26.6 (22.2, 28.7)	9.5 (3.6, 53.8)	9.8 (5.9, 16.4)	15.3 (7.9, 22.7)	13.6 (5.5, 26.3)	15.7 (3.6, 53.8)

B.						
Variable	VIT‐2763 60 mg QD (N = 8)	VIT‐2763 120 mg QD (N = 6)	VIT‐2763 60 mg BID (N = 6)	VIT‐2763 120 mg BID (N = 6)	Pooled placebo QD or BID (N = 8)	Total N = 34
Age, years, median (min, max)	22.5 (18, 62)	23.0 (19, 27)	33.5 (26, 60)	29.5 (18, 59)	23.5 (18, 31)	25.0 (18, 62)
Female sex, n (%)	6 (75)	4 (67)	4 (67)	3 (50)	7 (88)	24 (71)
White race, n (%)	8 (100)	6 (100)	4 (67)	5 (83)	6 (75)	29 (85)
Weight, kg, median (min, max)	73.7 (64.6, 81.9)	69.8 (62.7, 83.7)	65.2 (59.6, 72.5)	68.3 (53.5, 78.3)	64.6 (57.3, 73.6)	68.2 (53.5, 83.7)
BMI, kg/m^2^, median (min, max)	24.5 (21.8, 28.0)	23.1 (22.0, 29.3)	25.2 (20.1, 27.1)	22.0 (19.0, 24.4)	23.0 (19.6, 23.2)	23.1 (19.0, 29.3)
Serum ferritin, ng/mL, median (min, max)	53.0 (12, 169)	51.5 (14, 115)	38.5 (12, 183)	68.0 (9, 201)	45.5 (20, 58)	49.5 (9, 201)
TSAT, %, median (min, max)	29.1 (11.2, 47.2)	26.4 (15.5, 42.4)	21.4 (15.4, 25.6)	27.4 (6.4, 34.7)	28.6 (18.0, 47.3)	25.9 (6.4, 47.3)
Serum iron, μg/dL, median (min, max)	91.4 (44.2, 168.3)	88.4 (67.1, 142.5)	73.0 (59.8, 80.5)	98.1 (24.0, 120.7)	98.4 (63.7, 154.8)	86.7 (24.0, 168.3)
Serum transferrin, mg/dL, median (min, max)	231.0 (194, 281)	249.5 (214, 308)	243.0 (205, 298)	258.5 (235, 283)	249.5 (217, 324)	249.5 (194, 324)
Hemoglobin, g/dL, median (min, max)	13.8 (13.0, 17.4)	13.4 (12.2, 15.6)	14.4 (13.5, 15.6)	15.2 (12.1, 15.8)	13.5 (12.9, 15.0)	13.9 (12.1, 17.4)
Hepcidin, ng/mL, median (min, max)	25.9 (4.5, 44.3)	13.9 (3.0, 16.9)	13.1 (5.8, 27.2)	17.6 (3.5, 24.2)	10.0 (7.1, 24.3)	13.9 (3.0, 44.3)

Abbreviations: BID, twice daily; BMI, body mass index; N, number of participants dosed with each treatment (or any treatment as applicable); n, number of participants with characteristic; QD, once daily; TSAT, transferrin saturation.

### 3.2 Safety and tolerability

In the SAD cohorts, 23 AEs occurred in 15 out of 29 VIT‐2763‐treated participants (51.7%) and nine occurred in five out of nine placebo‐treated participants (55.6%). In the MAD cohorts, 75 AEs occurred in 24 out of 26 VIT‐2763‐treated participants (92.3%), and 21 occurred in five out of eight placebo‐treated participants (62.5%). Headache was the most commonly reported AE overall, occurring in 18.4% and 26.5% of participants in the SAD and MAD cohorts, respectively, and, at a similar frequency in the VIT‐2763 and placebo groups. The most common AEs and a complete list of all AEs are presented in Tables S3 and S4, respectively. There were no deaths or serious AEs, and no AEs led to discontinuation of the study drug. Most AEs (22/32 in the SAD phase; 68/96 in the MAD phase) were considered unrelated to the study drug (Table S5). Of the drug‐related AEs, one case of decreased blood iron in the MAD phase was considered to be probably/likely related to the study drug and all others were deemed as possibly related to the study drug. All AEs in the SAD phase and the majority (93/96) of AEs in the MAD phase were mild in severity (Table S5). Moderate AEs occurred in three participants (in the MAD phase); there were no AEs of severe or life‐threatening severity. The majority of AEs were transient and resolved without sequelae; at follow‐up, three AEs in the SAD phase and four AEs in the MAD phase, all of which were mild in severity, had not yet resolved. There were no clinically relevant changes from baseline in laboratory‐assessed safety parameters, vital signs, ECG results or physical examination.

### 3.3 Pharmacokinetic parameters

**Figure 1:**
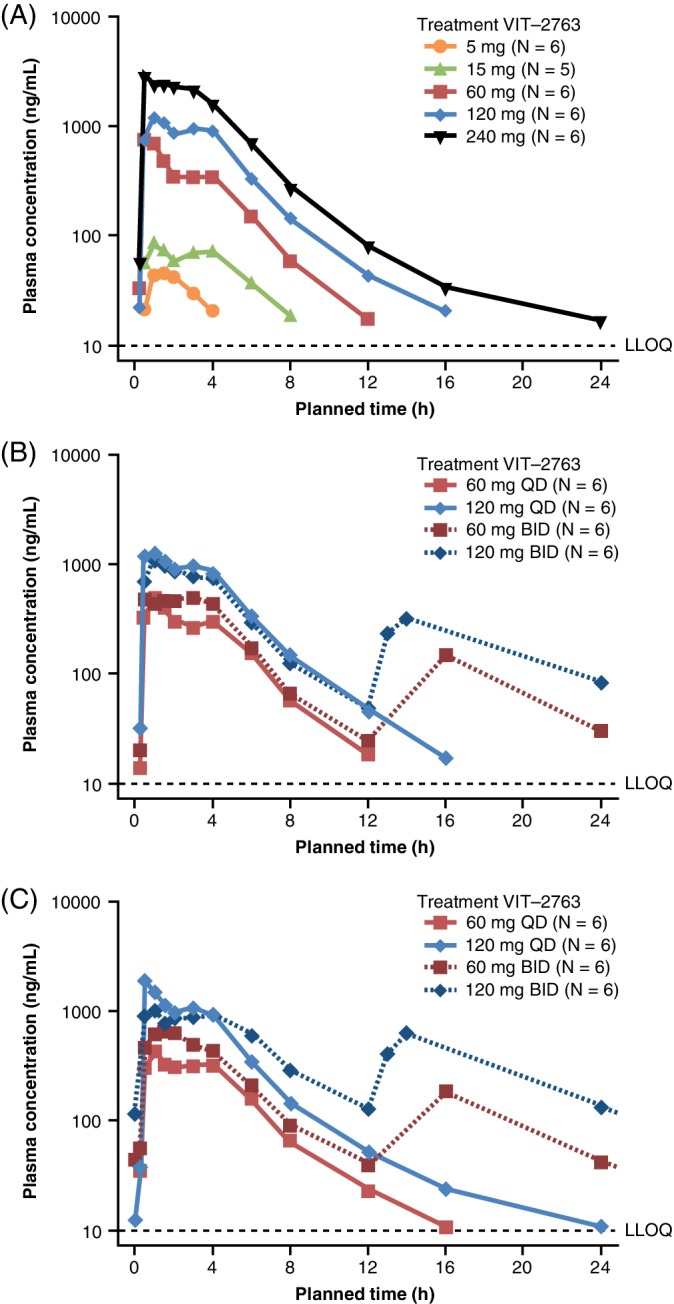
Plasma concentration─time profiles for VIT‐2763 for single‐ascending dose cohorts (A) and multiple‐ascending dose cohorts on Day 1 (B) and Day 7 (C) (per‐protocol population). Data shown are geometric mean values (semi‐logarithmic scale). Abbreviations: BID, twice daily; LLOQ, lower limit of quantification; PK, pharmacokinetic; QD, once daily. Morning doses of VIT‐2763 were administered after 10 hours of overnight fast. In the BID dosing cohorts, the evening dose was administered approximately 12 hours later, before dinner but after PK sampling. Fewer blood samples were taken following the evening dose compared with the morning dose due to logistical reasons

In the SAD cohorts, the initial oral absorption of VIT‐2763 was relatively fast, with detectable levels observed in most participants 15 to 30 minutes post‐dose. Across the VIT‐2763 cohorts in the SAD phase, the median T_max_ ranged from 0.50 to 3.00 hours and the geometric mean elimination T_1/2_ was 1.88 to 5.33 hours (Table 2). For the majority of participants who received single doses of ≥15 mg, a second peak or plateauing in the concentration─time profile occurred 3 to 4 hours post‐dose (Figure 1A). Elimination was monophasic following single doses of 5 mg, 15 mg or 60 mg, but biphasic for single doses of 120 mg or 240 mg (Figure 1A). A C_max_ exceeding 3805 ng/mL was observed in two participants following a single dose of 240 mg. Therefore, no participants in the study received higher doses.

**Table 2:** Pharmacokinetic parameters for single‐ascending dose cohorts (A) and multiple‐ascending dose cohorts (B)

A.					
Parameter	VIT‐2763 5 mg N = 6	VIT‐2763 15 mg N = 5	VIT‐2763 60 mg N = 6	VIT‐2763 120 mg N = 6	VIT‐2763 240 mg N = 6
C_max_ (ng/mL)	52.0 (39.2, 70.4)	98.1 (65.4, 115)	1049 (699, 1300)	1626 (1050, 2480)	3386 (2380, 4870)
T_max_ (h)	1.29 (1.00, 2.00)	3.00 (1.00, 4.00)	0.50 (0.50, 1.53)	1.25 (0.50, 4.00)	0.53 (0.50, 1.50)
T_1/2_ (h)	1.89 (1.48, 2.30) (N = 5)^a^	2.28 (1.72, 2.78) (N = 4)^a^	1.88 (1.79, 1.99) (N = 5)^a^	4.47 (2.19, 6.65)	5.33 (4.66, 6.22)
AUC_0‐last_ (ng h/mL)	145 (103, 196)	440 (353, 529)	2612 (1709, 3686)	5836 (4101, 7423)	12620 (9193, 18522)
AUC_0‐inf_ (ng h/mL)	217 (199, 240) (N = 3)^a^	520 (418, 570) (N = 4)^a^	2535 (1747, 3761) (N = 5)^a^	5928 (4165, 7472)	12758 (9355, 18664)
AUC_0‐12_ (ng h/mL)	NA	NA	2585 (1708, 3686)	5626 (4023, 7319)	12209 (8748, 17969)
CL/F (L/h)	23.1 (20.8, 25.2) (N = 3)^a^	28.8 (26.3, 35.9) (N = 4)^a^	23.7 (16.0, 34.3) (N = 5)^a^	20.2 (16.1, 28.8)	18.8 (12.9, 25.7)
V_z_/F (L)	67.7 (63.0, 77.8) (N = 3)^a^	95.0 (68.7, 140) (N = 4)^a^	64.3 (45.8, 94.0) (N = 5)^a^	131 (50.7, 244)	145 (86.4, 218)

B.					
Parameter	Analysis Day	VIT‐2763 60 mg QD (N = 6)	VIT‐2763 120 mg QD (N = 6)	VIT‐2763 60 mg BID (N = 6)	VIT‐2763 120 mg BID (N = 6)
C_max_ (ng/mL)	1	694 (251, 1030)	1720 (1100, 3070)	746 (562, 1000)	1777 (1420, 2430)
	7	581 (298, 1140)	2046 (1370, 2650)	916 (689, 1800)	1480 (900, 2690)
T_max_ (h)	1	1.00 (0.50, 3.00)	0.76 (0.52, 3.00)	0.75 (0.50, 4.00)	1.75 (0.50, 4.03)
	7	1.76 (0.50, 4.02)	0.50 (0.50, 1.50)	1.25 (0.50, 2.00)	1.00 (0.50, 4.00)
T_1/2_ (h)	1	2.07 (1.56, 2.47)	3.80 (1.96, 6.90)	2.29 (1.87, 3.36)	2.27 (1.98, 2.64)
	7	3.01 (1.95, 6.44)	5.26 (2.71, 7.60) (N = 5)^a^	2.61 (2.08, 3.04)	2.80 (2.47, 3.23)
AUC_0‐last_ (ng h/mL)	1	2153 (1252, 3275)	5975 (4380, 8472)	2814 (2420, 3672)	5453 (3366, 9777)
	7	2292 (1379, 4712)	6736 (5286, 8911)	3255 (2532, 4477)	6879 (5259, 9630)
AUC_0‐inf_ (ng h/mL)	1	2201 (1281, 3329)	6073 (4427, 8561)	2901 (2522, 3743)	5622 (3430, 10125)
	7	2356 (1436, 4771)	7178 (5344, 9060) (N = 5)^a^	3409 (2650, 4707)	7415 (5652, 10212)
AUC_0‐12_ (ng h/mL)	1	2141 (1255, 3173)	5790 (4296, 8192)	2816 (2423, 3674)	5457 (3368, 9785)
	7	NA	NA	3258 (2534, 4480)	6889 (5266, 9641)
AUC_0‐24_ (ng h/mL)	1	2199 (1280, 3322)	6010 (4421, 8475)	NA	NA
	7	2333 (1424, 4764)	6756 (5336, 8911)	NA	NA
CL/F (L/h)	1	27.3 (18.0, 46.8)	19.8 (14.0, 27.1)	20.7 (16.0, 23.8)	21.3 (11.9, 35.0)
	7	25.7 (12.6, 42.1)	17.0 (13.5, 22.5) (N = 5)^a^	18.4 (13.4, 23.7)	17.4 (12.4, 22.8)
V_z_/F (L)	1	81.2 (56.2, 118)	108 (63.0, 194)	68.3 (43.6, 101)	69.9 (39.6, 114)
V_ss_/F (L)	7	112 (41.6, 211)	129 (87.9, 188) (N = 5)^a^	69.2 (54.9, 91.0)	70.3 (50.7, 106)
C_avg_ (ng/mL)	7	97.2 (59.3, 199)	282 (222, 371)	271 (211, 373)	574 (439, 803)
C_min_ (ng/mL)	7	(<LLOQ, 13.3)	16.2 (<LLOQ, 19.4)	38.9 (30.8, 52.5)	129 (84.3, 172)
Rac (AUC)	7	1.06 (0.54, 2.42)	1.12 (0.95, 1.42)	1.16 (0.94, 1.63)	1.26 (0.99, 1.56)
Rac (C_max_)	7	0.84 (0.33, 1.61)	1.19 (0.61, 1.90)	1.23 (0.92, 1.80)	0.83 (0.60, 1.17)

Note: Data shown are geometric mean (range) (per‐protocol analysis). Numbers of participants are provided where data were not available for the whole population.

a Participants with suspected unreliable pharmacokinetic parameters as defined in the SAP were excluded from the descriptive statistics.

Note: For T_max_ the median (range) is presented instead of geometric mean (range).

Abbreviations: AUC, area under the plasma concentration‐time curve; AUC_0‐12_, area under the plasma concentration–time curve over the dosing interval (time 0 to 12 hours); AUC_0‐24_, area under the plasma concentration–time curve over the dosing interval (time 0 to 24 hours); AUC_0‐inf,_ area under the plasma concentration–time curve (time 0 to affinity); AUC_0‐last_, area under the plasma concentration–time curve (time 0 to time of last quantifiable concentration); BID, twice daily; C_avg_, average plasma concentration in the dosing interval; CL/F, apparent oral clearance; C_max_, maximum plasma concentration; C_min_, minimum plasma concentration; N, number of participants per treatment; NA, not applicable; QD, once daily; Rac, accumulation ratio; SAP, statistical analysis plan; T_1/2_, terminal phase half‐life; T_max_, time to maximum plasma concentration; V_ss_/F, apparent volume of distribution at terminal phase for steady‐state conditions; V_z_/F, apparent volume of distribution at terminal phase.

As in the SAD phase, a second peak in the concentration─time profiles was seen 3 to 4 hours post‐dose on Day 1 and 7 in the MAD cohorts (Figure 1B,C). No significant accumulation was observed after 7 days of repeated dosing for both once daily and twice daily dosing regimens (accumulation index values were 1.13 to 1.18). At Day 7 steady‐state, similar C_avg,ss_ was observed for a total daily dose of 120 mg, regardless of administration as a 120 mg once daily or 60 mg twice daily dose (282 and 271 ng/mL, respectively; Table 2). Across dosing regimens, the geometric mean elimination T_1/2_ was 2.07 to 3.80 hours on Day 1 and 2.61 to 5.26 hours on Day 7, following repeated dosing (Table 2). The stopping criterion C_max_ ≥3805 ng/mL was not reached after 7 days of dosing in any cohort in the MAD phase.

The correlation between individual exposure parameters (C_max_, AUC_0‐last_, AUC_0‐inf_ and AUC_0‐12_) and the received dose divided by the participant's body weight was assessed using scatter plots with a regression line. The R^2^ and P value suggest that the linear model relating PK parameters to dose/weight ratio fits well to the data (Figures S3 and S4A‐C).

### 3.4 Pharmacodynamic parameters

**Figure 2:**
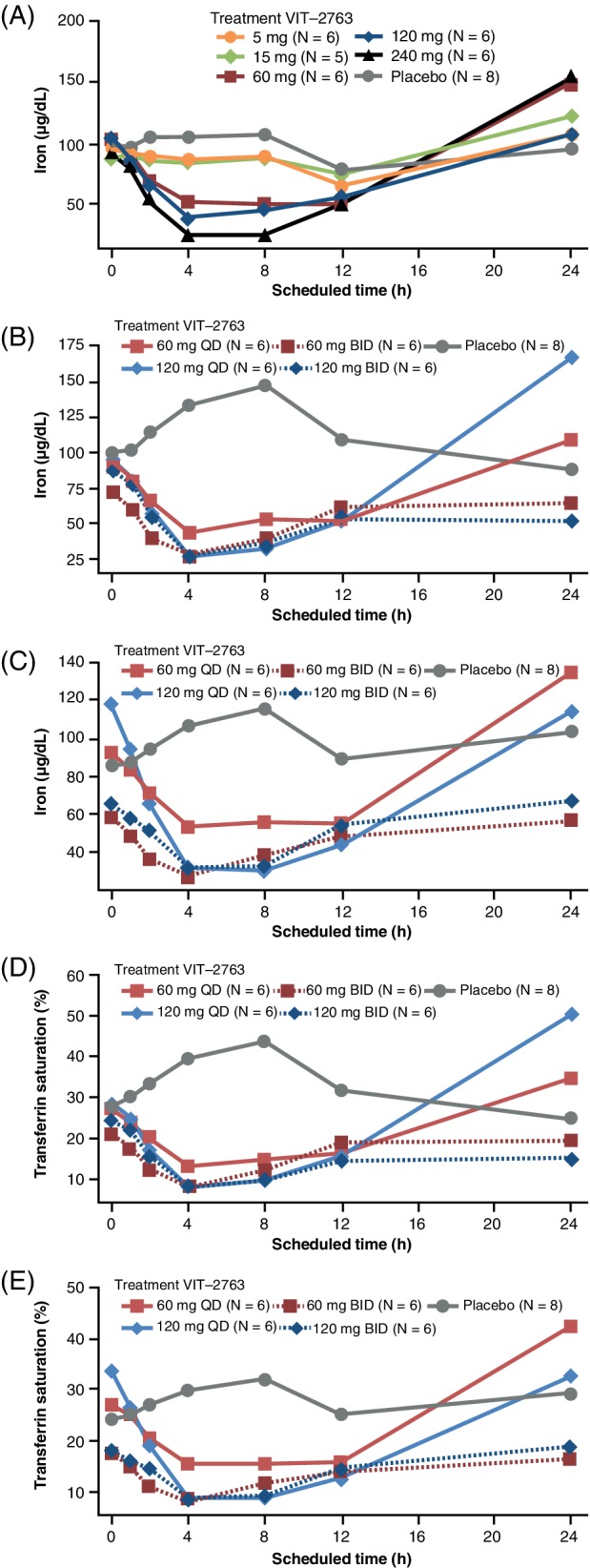
Mean serum iron vs time for single‐ascending dose cohorts (A) and multiple‐ascending dose cohorts on Day 1 (B) and Day 7 (C); and mean transferrin saturation vs time for multiple‐ascending dose cohorts at Day 1 (D) and Day 7 (E) (per‐protocol population). Abbreviations: BID, twice daily; QD, once daily

There was a temporary decrease in mean serum iron levels following the higher single doses of VIT‐2763 (60, 120 and 240 mg; Figure 2A), with minimum levels observed 4 or 8 hours post‐dose. For all single doses, mean serum iron levels rebounded to baseline or above by 24 hours post‐dose. Following all multiple doses of VIT‐2763, mean serum iron levels temporarily decreased, with minimum mean values being reached at 4 hours post‐dose on both Day 1 and Day 7 (Figure 2B,C). No temporary decrease in serum iron levels was observed following administration of placebo. All multiple doses of VIT‐2763 also led to a temporary decrease in mean calculated transferrin saturation, reaching a minimum mean value 4 hours post‐dose on both Day 1 and Day 7. No such decrease occurred with placebo (Figure 2D,E).

In the SAD and MAD phases, mean serum hepcidin peaked at 12 hours post‐dose in the placebo group. There was a shift in peaks following single doses of VIT‐2763 ≥60 mg at around VIT‐2763 T_max_, with maximum mean serum hepcidin levels observed at 2 or 4 hours post‐dose. There was a shift in peaks following multiple dosing with maximum mean serum hepcidin levels observed between 1 and 4 hours post‐dose on both Day 1 and Day 7. However, mean serum hepcidin levels appeared to decrease during the 7 days for all doses, except the 120 mg once daily dose level, which showed the most pronounced peak shift on Day 7 (Figure S5A‐C). Maximum mean serum hepcidin levels were observed at 2 or 4 hours post‐dose following single doses, and between 1 and 4 hours post‐dose following multiple doses on both Day 1 and Day 7. Levels returned to baseline values or below by 24 hours after single and multiple dosing of VIT‐2763 and in the placebo cohorts.

Following multiple dosing, there were no dose‐related effects on transferrin, erythropoietin or soluble transferrin receptor levels on Day 1 or 7 (Figures S6‐S8). There were also no relevant changes in the following measures: serum ferritin, mean volume of RBCs; mean RBC hemoglobin; mean RBC hemoglobin concentration; RBC distribution width; percentage of hypochromic RBCs; hemoglobin content of reticulocytes.

## 4 DISCUSSION

The results from this first‐in‐human study demonstrate that VIT‐2763, at single oral doses up to 240 mg, or multiple oral doses up to 120 mg twice daily, was well tolerated in healthy volunteers. There were no serious or severe AEs, or discontinuations due to AEs, and no discernible effects on any other safety parameters. After administration, VIT‐2763 was rapidly absorbed, showed no evidence of accumulation upon repeated dosing, and was associated with a decrease in serum iron levels. Together, these findings support the potential use of VIT‐2763 as a treatment for NTDT and other indications in which iron metabolism is involved.

Most participants had detectable levels of VIT‐2763 as early as 15 to 30 minutes post‐dose, with maximum levels reached between 30 minutes to 3 hours post‐dose. The human PK profile exhibited dose linearity, without relevant accumulation after 7 days of repeated dosing for both the once daily and twice daily dosing regimens; preclinical modeling work is ongoing to assess the PK‐PD relationship and confirm the preferred dosing schedule.

Single doses of VIT‐2763 ≥60 mg and all investigated multiple‐dose levels led to a rapid and prominent decrease in levels of circulating iron; no corresponding decreases were observed with placebo. This iron‐lowering PD effect is consistent with observations in a preclinical mouse model of NTDT and in wild‐type mice.^32^ In the current study, VIT‐2763 (all multiple‐dose levels) was also found to reduce transferrin saturation, an important finding since detectable levels of NTBI typically occur when transferrin saturation exceeds 75%.^35^ Hepatocytes and cardiomyocytes rapidly take up NTBI, which is thought to be responsible for toxicity to the liver and heart, the most critical organs damaged by iron‐loading disorders.^36^, ^37^ The PD effects of VIT‐2763 observed in the current study, in terms of reduced serum iron and transferrin saturation, suggest that VIT‐2763, via iron restriction, has the potential to improve erythropoiesis and anemia in patients with ineffective erythropoiesis, such as in NTDT, sickle cell disease and myelodysplastic syndrome. Also, VIT‐2763 may be able to reduce excessive iron absorption in hereditary hemochromatosis, and reduce excessive erythropoiesis in polycythemia vera. Of note, data from the current study indicate that VIT‐2763 dosing impacts the kinetics of hepcidin, shifting peak levels to an earlier time point, compared with the placebo group. This finding is surprising given that preclinical data show that in healthy mice, administration of VIT‐2763 leads to a rapid lowering of hepcidin transcription in the liver as a feedback response to declining blood iron levels (unpublished data). Clearly, the discrepancy between preclinical and clinical findings cannot easily be explained with the limited data from this Phase I study and warrants further investigation. The shift in peak levels was less pronounced after 7 days of multiple dosing, especially in the twice daily cohorts, where there was a near complete lack of hepcidin peak. No changes were observed in other hematologic biomarkers, which is as expected in a healthy population and over the short study period (only 1 week of dosing).

The gene therapy lentiglobin acts to increase hemoglobin levels and may provide a curative treatment for β‐thalassemia. However, the therapy is currently only approved for selected patients with TDT (those 12 years of age or over, who do not have a β0/β0 genotype, and for whom hematopoetic stem cell transplantation is appropriate but a suitable donor is lacking).^24^ Of the other treatments for β‐thalassemia that are currently in development, VIT‐2763 is the only orally administered agent that targets ferroportin. Other agents in development aim to reduce iron availability by mimicking hepcidin, or increasing the production of hepcidin. The synthetic hepcidin LJPC‐401 demonstrated iron‐lowering effects in Phase 1,^38^ and is now undergoing evaluation in Phase 2 trials for the treatment of iron overload in patients with TDT,^39^ and patients with hereditary hemochromatosis.^40^ The hepcidin peptidomimetic, PTG‐300, also demonstrated iron‐lowering effects in Phase 1,^41^ and is now being studied in a Phase 2 trial in patients with NTDT and TDT.^42^ Minihepcidins and siRNA targeting Tmprss6, both of which act by increasing the production of endogenous hepcidin, have been shown to improve erythropoiesis and anemia in mouse models of β‐thalassemia.^26^, ^27^, ^43^ By contrast, luspatercept, an activin receptor ligand trap, acts by promoting late‐stage erythroid differentiation, rather than restricting iron availability.^44^ Regulatory applications for the approval of luspatercept for the treatment of β‐thalassemia and myelodysplastic syndrome have been submitted in Europe and the US.^45^, ^46^

Unlike VIT‐2763, which is given orally, other agents in development are administered intravenously or subcutaneously. Parenterally administered drugs avoid first‐pass metabolism, can have a faster onset of action than orally administered medications, and may be beneficial if the patient is unlikely to adhere to self‐administered capsules or tablets. However, oral formulations may be more suitable for a pediatric population and do not require administration by a healthcare professional, which is costly as well as burdensome and inconvenient for patients. VIT‐2763 has a shorter half‐life than some of the parenteral formulations in development, such as luspatercept, which has a half‐life of 15 to 16 days.^44^ While a longer half‐life may allow for less frequent dosing, it also makes pharmacological effects more difficult to reverse, which may be problematic if, for example, there are safety concerns. In contrast, the dosing of orally administered compounds, such as VIT‐2763, can be adjusted daily if required.

As a life‐long disease, β‐thalassemia requires chronic treatment. Patients with TDT rely on frequent, regular blood transfusions throughout their life for survival, while those with NTDT may require occasional, or sometimes frequent, transfusions for defined periods of time.^8^, ^9^ Agents under development have the potential to reduce requirements for blood transfusions by increasing hemoglobin, and to thereby reduce the corresponding need for chelators, which are often associated with AEs.^15^, ^25^, ^47^, ^48^ Thus, new therapies may offer significant benefit to patients and also result in savings in terms of cost and resources.^25^ With the variety of treatments currently under development, the potential exists for combinations of agents with different mechanisms of action to be evaluated. In addition, agents may have beneficial effects in a wide range of diseases, including other conditions in which erythropoiesis is disrupted (such as sickle cell anemia and myelodysplastic syndrome), conditions where there is a chronic over‐absorption of iron (such as hemochromatosis), and conditions involving excessive proliferation of RBCs (such as polycythemia vera).

Strengths of the current study include the wide range of safety, PK and PD endpoints assessed, both following single and multiple dosing which will support and inform the design of future trials. A limitation of the study is that blood samples for PK and PD analysis were not taken between 12 and 24 hours post‐dose, which may have impacted some observations, particularly with regards to serum iron in the twice daily dosing cohorts. Another potential limitation is that plasma VIT‐2763 PK parameters were calculated using non‐compartmental analysis; compartmental analytic techniques may have produced different results.

## 5 CONCLUSION

When VIT‐2763, an oral ferroportin inhibitor, was administered at single oral doses up to 240 mg, or multiple oral doses up to 120 mg twice daily, it had a comparable safety profile to the placebo, and was well tolerated in healthy volunteers. VIT‐2763 achieved rapid and prominent decreases in circulating iron levels, consistent with the iron‐lowering PD effect observed in preclinical models, and also reduced transferrin saturation. Thus, results from the current study suggest that limiting iron availability for erythropoiesis via inhibition of ferroportin activity may be a potential approach to treating NTDT, and other indications that involve ineffective erythropoiesis, excessive iron absorption or excessive erythropoiesis. Supported by these findings, clinical studies of VIT‐2763 in patients with ineffective erythropoiesis are warranted.

## CONFLICT OF INTEREST

F.R., B.R., T.H., U.‐M.G. and F.D. are all employees of Vifor. J.J.v.L. is an employee of PRA Health Sciences, Early Development Services, Groningen, Netherlands.

## AUTHOR CONTRIBUTIONS

All authors contributed to the research, conception and design of the study, data analysis and interpretation. All authors reviewed and revised drafts of the manuscript and approved the final draft for submission.

## Supporting information


**Appendix S1**: Supporting Information

## References

[1] Camaschella C . Iron and hepcidin: a story of recycling and balance. Hematology Am Soc Hematol Educ Program. 2013;2013:1‐8. 24319154 10.1182/asheducation-2013.1.1

[2] Tanno T , Miller JL . Iron loading and overloading due to ineffective erythropoiesis. Adv Hematol. 2010;2010:358283. 20467559 10.1155/2010/358283 PMC2868182

[3] Coffey R , Ganz T . Iron homeostasis: An anthropocentric perspective. J Biol Chem. 2017;292(31):12727‐12734. 28615456 10.1074/jbc.R117.781823 PMC5546013

[4] Kohne E . Hemoglobinopathies: clinical manifestations, diagnosis and treatment. Dtsch Arztebl Int. 2011;108(31–32):532‐540. 21886666 10.3238/arztebl.2011.0532 PMC3163784

[5] Cappellini MD , Motta I . New therapeutic targets in transfusion‐dependent and ‐independent thalassemia. Hematology Am Soc Hematol Educ Program. 2017;2017(1):278‐283. 29222267 10.1182/asheducation-2017.1.278 PMC6142569

[6] Viprakasit V , Ekwattanakit S . Clinical classification, screening and diagnosis for thalassemia. Hematol Oncol Clin North Am. 2018;32(2):193‐211. 29458726 10.1016/j.hoc.2017.11.006

[7] Oikonomidou PR , Casu C , Rivella S . New strategies to target iron metabolism for the treatment of beta thalassemia. Ann N Y Acad Sci. 2016;1368(1):162‐168. 26919168 10.1111/nyas.13018 PMC5271577

[8] Rachmilewitz EA , Giardina PJ . How I treat thalassemia. Blood. 2011;118(13):3479‐3488. 21813448 10.1182/blood-2010-08-300335

[9] Weatherall DJ . The definition and epidemiology of non‐transfusion‐dependent thalassemia. Blood Rev. 2012;26(Suppl 1):S3‐S56. 22631040 10.1016/S0268-960X(12)70003-6

[10] Origa R , Galanello R , Ganz T , et al. Liver iron concentrations and urinary hepcidin in beta‐thalassemia. Haematologica. 2007;92(5):583‐588. 17488680 10.3324/haematol.10842

[11] Taher AT , Weatherall DJ , Cappellini MD . Thalassaemia. Lancet. 2018;391(10116):155‐167. 28774421 10.1016/S0140-6736(17)31822-6

[12] Taher AT , Musallam KM , Cappellini MD . Thalassaemia intermedia: an update. Mediterr J Hematol Infect Dis. 2009;1(1):e2009004. 21415986 10.4084/MJHID.2009.004 PMC3033165

[13] Musallam KM , Taher AT , Rachmilewitz EA . Beta‐thalassemia intermedia: a clinical perspective. Cold Spring Harb Perspect Med. 2012;2(7):a013482. 22762026 10.1101/cshperspect.a013482 PMC3385943

[14] Taher A , Musallam KM , El Rassi F , et al. Levels of non‐transferrin‐bound iron as an index of iron overload in patients with thalassaemia intermedia. Br J Haematol. 2009;146(5):569‐572. 19604239 10.1111/j.1365-2141.2009.07810.x

[15] Shander A , Cappellini MD , Goodnough LT . Iron overload and toxicity: the hidden risk of multiple blood transfusions. Vox Sang. 2009;97(3):185‐197. 19663936 10.1111/j.1423-0410.2009.01207.x

[16] Camaschella C , Nai A . Ineffective erythropoiesis and regulation of iron status in iron loading anaemias. Br J Haematol. 2016;172(4):512‐523. 26491866 10.1111/bjh.13820

[17] Cao A , Galanello R . Beta‐thalassemia. Genet Med. 2010;12(2):61‐76. 20098328 10.1097/GIM.0b013e3181cd68ed

[18] Galanello R , Origa R . Beta‐thalassemia. Orphanet J Rare Dis. 2010;5:11. 20492708 10.1186/1750-1172-5-11 PMC2893117

[19] Breda L , Casu C , Gardenghi S , et al. Therapeutic hemoglobin levels after gene transfer in beta‐thalassemia mice and in hematopoietic cells of beta‐thalassemia and sickle cells disease patients. PLoS One. 2012;7(3):e32345. 22479321 10.1371/journal.pone.0032345 PMC3314006

[20] May C , Rivella S , Callegari J , et al. Therapeutic haemoglobin synthesis in beta‐thalassaemic mice expressing lentivirus‐encoded human beta‐globin. Nature. 2000;406(6791):82‐86. 10894546 10.1038/35017565

[21] Imren S , Payen E , Westerman KA , et al. Permanent and panerythroid correction of murine beta thalassemia by multiple lentiviral integration in hematopoietic stem cells. Proc Natl Acad Sci U S A. 2002;99(22):14380‐14385. 12391330 10.1073/pnas.212507099 PMC137892

[22] Cavazzana‐Calvo M , Payen E , Negre O , et al. Transfusion independence and HMGA2 activation after gene therapy of human beta‐thalassaemia. Nature. 2010;467(7313):318‐322. 20844535 10.1038/nature09328 PMC3355472

[23] Thompson AA , Walters MC , Kwiatkowski J , et al. Gene therapy in patients with transfusion‐dependent beta‐thalassemia. N Engl J Med. 2018;378(16):1479‐1493. 29669226 10.1056/NEJMoa1705342

[24] bluebird bio. bluebird bio announces EU conditional marketing authorization for ZYNTEGLO™ (autologous CD34+ cells encoding βA‐T87Q‐globin gene) gene therapy for patients 12 years and older with transfusion‐dependent β‐thalassemia who do not have β0/β0 genotype. 2019; http://investor.bluebirdbio.com/news-releases/news-release-details/bluebird-bio-announces-eu-conditional-marketing-authorization. Accessed July 10, 2019.

[25] Porter J . Beyond transfusion therapy: new therapies in thalassemia including drugs, alternate donor transplant, and gene therapy. Hematology Am Soc Hematol Educ Program. 2018;2018(1):361‐370. 30504333 10.1182/asheducation-2018.1.361 PMC6245990

[26] Casu C , Oikonomidou PR , Chen H , et al. Minihepcidin peptides as disease modifiers in mice affected by beta‐thalassemia and polycythemia vera. Blood. 2016;128(2):265‐276. 27154187 10.1182/blood-2015-10-676742 PMC4946204

[27] Schmidt PJ , Toudjarska I , Sendamarai AK , et al. An RNAi therapeutic targeting Tmprss6 decreases iron overload in Hfe(−/−) mice and ameliorates anemia and iron overload in murine beta‐thalassemia intermedia. Blood. 2013;121(7):1200‐1208. 23223430 10.1182/blood-2012-09-453977 PMC3655736

[28] Ward DM , Kaplan J . Ferroportin‐mediated iron transport: expression and regulation. Biochim Biophys Acta. 2012;1823(9):1426‐1433. 22440327 10.1016/j.bbamcr.2012.03.004 PMC3718258

[29] Schmidt PJ , Fleming MD . Modulation of hepcidin as therapy for primary and secondary iron overload disorders: preclinical models and approaches. Hematol Oncol Clin. 2014;28(2):387‐401. 10.1016/j.hoc.2013.11.004 PMC3942790 24589273

[30] Canonne‐Hergaux F , Donovan A , Delaby C , Wang HJ , Gros P . Comparative studies of duodenal and macrophage ferroportin proteins. Am J Physiol Gastrointest Liver Physiol. 2006;290(1):G156‐G163. 16081760 10.1152/ajpgi.00227.2005

[31] Rossi E . Hepcidin‐the iron regulatory hormone. Clin Biochem Rev. 2005;26(3):47‐49. 16450011 PMC1240030

[32] Manolova V , Nyffenegger N , Flace A , et al. Oral ferroportin inhibitor ameliorates ineffective erythropoiesis in a model of β‐thalassemia. J Clin Invest. 2019 10.1172/JCI129382. [Epub ahead of print] PMC6934209 31638596

[33] Porter J. Iron restricted erythropoiesis. Paper presented at: the European School of Haematology international conference 2019: Erythropoiesis control and ineffective erythropoiesis: from bench to bedside; 15–17 March, 2019; Budapest, Hungary.

[34] European Medicines Agency . Guideline on strategies to identify and mitigate risks for first‐in‐human and early clinical trials with investigational medicinal products. 2017; https://www.ema.europa.eu/en/documents/scientific-guideline/guideline-strategies-identify-mitigate-risks-first-human-early-clinical-trials-investigational_en.pdf. Accessed March 4, 2019. 10.1111/bcp.13550 PMC6005602 29451320

[35] Garbowski MW , Ma Y , Fucharoen S , Srichairatanakool S , Hider R , Porter JB . Clinical and methodological factors affecting non‐transferrin‐bound iron values using a novel fluorescent bead assay. Transl Res. 2016;177:19‐30. e15. 27344508 10.1016/j.trsl.2016.05.005 PMC5110642

[36] Piga A , Longo F , Duca L , et al. High nontransferrin bound iron levels and heart disease in thalassemia major. Am J Hematol. 2009;84(1):29‐33. 19006228 10.1002/ajh.21317

[37] Anderson GJ . Mechanisms of iron loading and toxicity. Am J Hematol. 2007;82(12 Suppl):1128‐1131. 17963252 10.1002/ajh.21075

[38] Porter J , Kowdley K , Taher A , et al. Effect of Ljpc‐401 (synthetic human hepcidin) on iron parameters in healthy adults. Blood. 2018;132:2336.

[39] http://clinicaltrials.gov. A study with LJPC‐401 for the treatment of myocardial iron overload in adult patients with transfusion‐dependent beta thalassemia. 2019; https://clinicaltrials.gov/ct2/show/NCT03381833?term=LJPC-401&rank=2. Accessed May 14, 2019.

[40] http://clinicaltrials.gov. A study of LJPC‐401 for the treatment of iron overload in adult patients with hereditary hemochromatosis. 2019; https://clinicaltrials.gov/ct2/show/NCT03395704?term=LJPC-401&rank=1. Accessed May 14, 2019.

[41] Protagonist Therapeutics. Protagonist announces phase 1 and pre‐clinical data on hepcidin mimetic PTG‐300 presented at European Hematology Association Annual Meeting. 2018; http://investors.protagonist-inc.com/news-releases/news-release-details/protagonist-announces-phase-1-and-pre-clinical-data-hepcidin. Accessed May 14, 2019.

[42] http://clinicaltrials.gov. Study of PTG‐300 in non‐transfusion dependent and transfusion‐dependent beta‐thalassemia subjects with chronic anemia (TRANSCEND). 2019; https://clinicaltrials.gov/ct2/show/NCT03802201?term=PTG-300&rank=1. Accessed May 14, 2019.

[43] Guo S , Casu C , Gardenghi S , et al. Reducing TMPRSS6 ameliorates hemochromatosis and beta‐thalassemia in mice. J Clin Invest. 2013;123(4):1531‐1541. 23524968 10.1172/JCI66969 PMC3613931

[44] Attie KM , Allison MJ , McClure T , et al. A phase 1 study of ACE‐536, a regulator of erythroid differentiation, in healthy volunteers. Am J Hematol. 2014;89(7):766‐770. 24715706 10.1002/ajh.23732 PMC4173124

[45] Acceleron Pharma. Celgene Corporation and Acceleron Pharma announce submission of Luspatercept biologics license application to U.S. FDA. 2019; https://ir.celgene.com/press-releases/press-release-details/2019/Celgene-Corporation-and-Acceleron-Pharma-Announce-Submission-of-Luspatercept-Biologics-License-Application-to-US-FDA/default.aspx. Accessed June 17, 2019.

[46] Celgene. Celgene Corporation and Acceleron Pharma announce submission of Luspatercept marketing authorization application to the European Medicines Agency (EMA) for MDS and beta‐thalassemia. 2019; http://investor.acceleronpharma.com/news-releases/news-release-details/celgene-corporation-and-acceleron-pharma-announce-submission-0. Accessed June 17, 2019.

[47] Kontoghiorghe CN , Kontoghiorghes GJ . Efficacy and safety of iron‐chelation therapy with deferoxamine, deferiprone, and deferasirox for the treatment of iron‐loaded patients with non‐transfusion‐dependent thalassemia syndromes. Drug Des Dev Ther. 2016;10:465‐481. 10.2147/DDDT.S79458 PMC4745840 26893541

[48] Di Maggio R , Maggio A . The new era of chelation treatments: effectiveness and safety of 10 different regimens for controlling iron overloading in thalassaemia major. Br J Haematol. 2017;178(5):676‐688. 28439891 10.1111/bjh.14712

