# Supplementary material for: Oral ferroportin inhibitor VIT‐2763: First‐in‐human, phase 1 study in healthy volunteers
Source: Am J Hematol. 2019 Nov 19;95(1):68–77. doi: 10.1002/ajh.25670 (PMC6916274; doi:10.1002/ajh.25670)
Supplement: Supplementary file 1 — Appendix S1: Supporting Information [file AJH-95-68-s001.docx]

**Supplemental data for:**

**Oral ferroportin inhibitor VIT-2763: first-in-human, phase 1 study in healthy volunteers**

Frank Richard, Jan Jaap van Lier, Bernard Roubert, Teba Haboubi, Udo-Michael Göhring and Franz Dürrenberger

**Corresponding author:** Frank Richard

**Phone number:** +41 58 851 83 81

**Email:** frank.richard@viforpharma.com

**Supplementary tables**

**Supplementary Table 1. Full inclusion/exclusion criteria.**

| **Inclusion criteria** |
| --- |
| Male or female participants, 18-65 years of age inclusive. |
| Non-smokers, former smokers, or current low-rate daily smokers (≤ 5 cigarettes or equivalent per day). |
| BMI between 18.5 and 29.9 kg/m^2^ and body weight between 50 and 100 kg. |
| Good general health, as determined by medical history, physical examination, vital signs, 12-lead ECG and safety laboratory testing. |
| Ability to understand the requirements of the study and abide by the study restrictions, and agreement to return for the required assessments. |
| Written informed consent prior to any study-specific procedures (including screening procedures). |
| **Exclusion criteria** |
| Any history of iron storage diseases such as hemochromatosis. |
| Any history or clinical findings of iron utilization disorders such as sideroblastic anemia. |
| Known hemoglobinopathy (e.g. thalassemia). |
| History of intravenous iron therapy, erythropoiesis stimulating agent therapy and/or blood transfusion in 3 months prior to screening, and/or excessive oral iron or oral iron-containing products intake exceeding the recommended dietary allowances for iron in adults (8 mg/day and 18 mg/day in males and females, respectively) on weekly average in the 4 weeks prior to randomization. |
| Blood draw of ≥ 20 to < 200 ml within 2 weeks, ≥ 200 to < 400 ml within 4 weeks, or ≥ 400 ml within 12 weeks (male) or within 16 weeks (female) prior to screening. |
| Need for blood transfusion or Hb < 13 g/dl (8.1 mmol/l) and < 12 g/dl (7.5 mmol/l) for male and female participants, respectively, at screening. |
| Serum ferritin < 16 ng/ml or > 300 ng/ml in females, and < 30 ng/ml or > 600 ng/ml in males. |
| A laboratory value for hsCRP ≥ 3.0 mg/l, if suspected indicative for inflammation. |
| Chronic liver disease and/or ALT, AST or GGT > 1.5x ULN at screening. |
| Serum creatinine > 1.5x ULN at screening. |
| Any history or clinical finding of cardiac disorders, such as clinically relevant cardiac arrhythmia, cardiomyopathy, coronary disease, valve disorder, or heart failure. |
| Any clinically relevant abnormal 12-lead ECG finding during screening or prior to randomization (as deemed by the PI) including, but not limited to any of the following:   - - PR interval > 200 ms or < 120 ms   - Evidence or history of second- or third-degree AV block   - QTcF interval ≥ 430 ms for male and ≥ 450 ms for female participants at screening (single reading) and at pre-dose (mean of triplicate readings taken in intervals of approximately 2 minutes)   - QRS interval > 104 ms. |
| Family history of long‐QT syndrome or sudden death without a preceding diagnosis of a condition that could be causative of sudden death (such as known coronary artery disease, congestive heart failure or terminal cancer). |
| Use of any medication that prolongs the QT/QTc interval or the PR/QRS interval, within 6 weeks prior to screening. |
| Clinically relevant deviations in the clinical laboratory results, including RBC count and WBC count, as judged by the PI. |
| Known history, and/or positive result on screening for HBsAg, HBV, HCV or HIV infection, or AIDS, or other active infection. |
| Use of any prohibited medication prior to screening. |
| Known sensitivity to any of the study products to be administered. |
| Participation in any other investigational device or drug study within 30 days prior to screening. Participation in protein therapeutic studies within 3 months prior to screening. |
| Pregnant (e.g. positive pregnancy test) or breast-feeding women. |
| Female participants of childbearing potential and male participants who did not agree to use highly effective methods of contraception during the study and for 3 months after the last dose of study medication. The highly effective methods of contraception included: intrauterine device, bilateral tubal occlusion, vasectomized partner, combined continuous hormonal contraception by the female participant and male partner using a condom with spermicide (or adequate and approved alternative such as Contragel Green), or sexual abstinence. |
| History of drug or alcohol abuse within 2 years prior to screening, positive screen for drug abuse or alcohol at screening or admission. |
| History of more than moderate alcohol consumption (> 200 g alcohol per week, equivalent to 3–4 litres of normal beer of approximately 5% volume). |
| Consumption of > 5 cups of xanthine- (coffee/tea) containing beverages per day within the 72 hours prior to screening and baseline. |
| Strenuous physical exercise (including competitive sport) within the 72 hours prior to screening and baseline. |
| Unable to swallow a placebo Size 0 capsule at screening. |
| Significant medical condition(s), anticipated need for major surgery during the study, or any other kind of disorder that may be associated with increased risk to the participant, or may interfere with study assessments, outcomes, or the ability to provide written informed consent or comply with study procedures, in the PI’s opinion. |
| Vulnerable participants, e.g. participants kept in detention, protected adults under guardianship, trusteeship and soldiers or participants committed to an institution by governmental or juridical order. |
| Any employee or their close relatives of Vifor Pharma, or of a Contract Research Organization or a study site involved in the study. |

AIDS, acquired immunodeficiency syndrome; ALT, alanine transaminase; AST, aspartate transaminase; AV, atrioventricular; BMI, body mass index; ECG, electrocardiogram; GGT, gamma glutamyl transpeptidase; Hb, hemoglobin; HBsAg, hepatitis B surface antigen; HBV, hepatitis B virus; HCV, hepatitis C virus; HIV, human immunodeficiency virus; hsCRP, high sensitivity C-reactive protein; PI, principal investigator; RBC, red blood cell; ULN, upper limit of normal; WBC, white blood cell

**Supplementary Table 2. Study endpoints.**

| **Primary endpoints** |
| --- |
| Safety endpoints: |
| Incidence of AEs. |
| Incidence SAEs. |
| Changes in vital signs (blood pressure, pulse rate, body temperature and respiratory rate). |
| Clinical laboratory safety tests (hematology, serum chemistry, coagulation, and urinalysis). |
| 12-lead ECG. |
| Cardiac telemetry. |
| Physical examination findings. |
| **Secondary endpoints** |
| Pharmacokinetic endpoints: |
| Plasma concentrations of VIT-2763 to determine the following parameters following single or multiple dosing: C_max_, T_max_, T_1/2_, T_z_, λ_z_, AUC_0–t_, AUC_0–last_, AUC_0–inf_, CL/F, V_z_/F. |
| Plasma concentrations of VIT-2763 to determine the following SS parameters following multiple dosing: V_ss_/F, C_ss,max_, T_ss,max_, C_ss,min_, C_ss,avg_. |
| Pharmacodynamic endpoints: |
| SAD phase: Serum iron and hepcidin. |
| MAD phase: Serum iron, hepcidin, calculated TSAT, serum ferritin, serum transferrin, EPO, and soluble transferrin receptor. |
| MAD phase: Additional pharmacodynamic markers: mean corpuscular volume, mean corpuscular hemoglobin, mean corpuscular hemoglobin concentration, red blood cell distribution width, percentage of hypochromic red blood cells, hemoglobin content of reticulocytes. |

AE, adverse event; AUC_0–t_, area under the plasma concentration–time curve over the dosing interval; AUC_0–inf_, area under the plasma concentration–time curve (time 0 to infinity); AUC_0–last_, area under the concentration–time curve (time 0 to time of last quantifiable measure); CL/F, apparent oral clearance; C_max_, maximum plasma concentration; C_ss,avg_, average plasma concentration in the dosing interval for steady-state conditions; C_ss,max_, maximum plasma concentration for steady-state conditions; C_ss,min_, minimum plasma concentration for steady-state conditions; ECG, electrocardiogram; EPO, erythropoietin; MAD, multiple-ascending dose; SAD, single-ascending dose; SAE, serious adverse event; SS, steady-state; T_1/2_, terminal phase half-life; T_max_, time to maximum plasma concentration; TSAT, transferrin saturation; T_ss, max_, time to maximum plasma concentration for steady-state conditions; T_z_, time of last measurable concentration; V_z_/F, apparent volume of distribution at terminal phase; V_ss_/F, apparent volume of distribution at terminal phase for steady-state; λ_z_, terminal elimination rate constant

**Supplementary Table 3. Summary of most common AEs (occurred in ≥5% of participants in total) for single-ascending dose cohorts (A) and multiple-ascending dose cohorts (B) (safety population).**

A.

| Preferred Term | VIT­2763 5 mg N=6 E/n (%) | | VIT­2763 15 mg N=5  E/n (%) | | VIT­2763 60 mg N=6  E/n (%) | | VIT­2763 120 mg N=6  E/n (%) | | VIT­2763 240 mg N=6 E/n (%) | | Pooled  placebo N=9  E/n (%) | | Total N=38  E/n (%) |
| --- | --- | --- | --- | --- | --- | --- | --- | --- | --- | --- | --- | --- | --- |
| ANY ADVERSE EVENTS | 3/2 (33) | | 4/3 (60) | | 4/3 (50) | | 5/3 (50) | | 7/4 (67) | | 9/5 (56) | | 32/20 (53) |
| Headache | 1/1 (17) | | 1/1 (20) | | 1/1 (17) | | 1/1 (17) | | 1/1 (17) | | 2/2 (22) | | 7/7 (18) |
| Somnolence | 0/0 (0) | | 1/1 (20) | | 1/1 (17) | | 0/0 (0) | | 1/1 (17) | | 2/2 (22) | | 5/5 (13) |
| Constipation | 0/0 (0) | | 1/1 (20) | | 0/0 (0) | | 2/2 (33) | | 0/0 (0) | | 0/0 (0) | | 3/3 (8) |
| Dizziness | 0/0 (0) | | 0/0 (0) | | 0/0 (0) | | 0/0 (0) | | 1/1 (17) | | 1/1 (11) | | 2/2 (5) |
| Neutrophil count decreased | 1/1 (17) | | 0/0 (0) | | 0/0 (0) | | 1/1 (17) | | 0/0 (0) | | 0/0 (0) | | 2/2 (5) |
| White blood cell count decreased | 1/1 (17) | | 0/0 (0) | | 0/0 (0) | | 1/1 (17) | | 0/0 (0) | | 0/0 (0) | | 2/2 (5) |
| Nasopharyngitis | 0/0 (0) | | 0/0 (0) | | 2/2 (33) | | 0/0 (0) | | 0/0 (0) | | 0/0 (0) | | 2/2 (5) |
|  |  |  | |  | |  | |  | |  | |  | |

B.

| Preferred Term | VIT­2763 60 QD N=8  E/n (%) | VIT­2763 120 mg QD N=6  E/n (%) | VIT­2763 60 mg BID N=6  E/n (%) | VIT­2763 120 mg BID N=6  E/n (%) | Pooled placebo QD or BID N=8  E/n (%) | Total N=34  E/n (%) |
| --- | --- | --- | --- | --- | --- | --- |
| ANY ADVERSE EVENTS | 21/8 (100) | 23/5 (83) | 11/5 (83) | 20/6 (100) | 21/5 (63) | 96/29 (85) |
| Headache | 4/3 (38) | 3/2 (33) | 1/1 (17) | 2/1 (17) | 3/2 (25) | 13/9 (26) |
| Medical device site reaction* | 0/0 (0) | 1/1 (17) | 1/1 (17) | 4/4 (67) | 1/1 (13) | 7/7 (21) |
| White blood cell count decreased | 1/1 (13) | 2/2 (33) | 0/0 (0) | 1/1 (17) | 2/2 (25) | 6/6 (18) |
| Neutrophil count decreased | 0/0 (0) | 1/1 (17) | 0/0 (0) | 2/2 (33) | 2/2 (25) | 5/5 (15) |
| Dizziness | 3/3 (38) | 0/0 (0) | 0/0 (0) | 0/0 (0) | 2/1 (13) | 5/4 (12) |
| Somnolence | 0/0 (0) | 2/2 (33) | 0/0 (0) | 0/0 (0) | 2/2 (25) | 4/4 (12) |
| Nausea | 0/0 (0) | 2/2 (33) | 0/0 (0) | 1/1 (17) | 2/1 (13) | 5/4 (12) |
| Myalgia | 1/1 (13) | 0/0 (0) | 0/0 (0) | 2/1 (17) | 2/2 (25) | 5/4 (12) |
| Abdominal pain | 0/0 (0) | 2/2 (33) | 0/0 (0) | 0/0 (0) | 1/1 (13) | 3/3 (9) |
| Fatigue | 0/0 (0) | 1/1 (17) | 0/0 (0) | 2/2 (33) | 0/0 (0) | 3/3 (9) |
| Abdominal distension | 0/0 (0) | 0/0 (0) | 1/1 (17) | 1/1 (17) | 0/0 (0) | 2/2 (6) |
| Diarrhea | 1/1 (13) | 1/1 (17) | 0/0 (0) | 0/0 (0) | 0/0 (0) | 2/2 (6) |
| Dysmenorrhea | 1/1 (13) | 1/1 (17) | 0/0 (0) | 0/0 (0) | 0/0 (0) | 2/2 (6) |
| Epistaxis | 2/2 (25) | 0/0 (0) | 0/0 (0) | 0/0 (0) | 0/0 (0) | 2/2 (6) |
| Oropharyngeal pain | 1/1 (13) | 1/1 (17) | 0/0 (0) | 0/0 (0) | 0/0 (0) | 2/2 (6) |
| Nasopharyngitis | 0/0 (0) | 0/0 (0) | 2/2 (33) | 0/0 (0) | 0/0 (0) | 2/2 (6) |
| Decreased appetite | 0/0 (0) | 2/2 (33) | 0/0 (0) | 0/0 (0) | 0/0 (0) | 2/2 (6) |

*Relates to medical devices/procedures (such as ECG electrodes reactions, or others) rather than the oral administration of the test drug

%=Number of events (E) as a percentage of number of participants that experienced an AE in this category (n); N, number of participants exposed per treatment

AE, adverse event; BID, twice daily; QD, once daily

**Supplementary Table 4. All AEs reported in single-ascending dose cohorts (A) and multiple-ascending dose cohorts (B) (safety population).**

A.

| System Organ Class  Preferred Term | VIT­2763 5 mg N=6  E/n (%) | VIT­2763 15 mg N=5 E/n (%) | VIT­2763 60 mg N=6  E/n (%) | VIT­2763 120 mg N=6 E/n (%) | VIT­2763 240 mg N=6 E/n (%) | Pooled  placebo N=9  E/n (%) | Total N=38  E/n (%) |
| --- | --- | --- | --- | --- | --- | --- | --- |
| ANY ADVERSE EVENTS | 3/2 (33) | 4/3 (60) | 4/3 (50) | 5/3 (50) | 7/4 (67) | 9/5 (56) | 32/20 (53) |
| NERVOUS SYSTEM DISORDERS | 1/1 (17) | 2/2 (40) | 2/2 (33) | 1/1 (17) | 3/3 (50) | 5/4 (44) | 14/13 (34) |
| Headache | 1/1 (17) | 1/1 (20) | 1/1 (17) | 1/1 (17) | 1/1 (17) | 2/2 (22) | 7/7 (18) |
| Somnolence | 0/0 (0) | 1/1 (20) | 1/1 (17) | 0/0 (0) | 1/1 (17) | 2/2 (22) | 5/5 (13) |
| Dizziness | 0/0 (0) | 0/0 (0) | 0/0 (0) | 0/0 (0) | 1/1 (17) | 1/1 (11) | 2/2 (5) |
| GASTROINTESTINAL DISORDERS | 0/0 (0) | 1/1 (20) | 0/0 (0) | 2/2 (33) | 1/1 (17) | 2/2 (22) | 6/6 (16) |
| Constipation | 0/0 (0) | 1/1 (20) | 0/0 (0) | 2/2 (33) | 0/0 (0) | 0/0 (0) | 3/3 (8) |
| Diarrhea | 0/0 (0) | 0/0 (0) | 0/0 (0) | 0/0 (0) | 0/0 (0) | 1/1 (11) | 1/1 (3) |
| Gingival pain | 0/0 (0) | 0/0 (0) | 0/0 (0) | 0/0 (0) | 0/0 (0) | 1/1 (11) | 1/1 (3) |
| Nausea | 0/0 (0) | 0/0 (0) | 0/0 (0) | 0/0 (0) | 1/1 (17) | 0/0 (0) | 1/1 (3) |
| INVESTIGATIONS | 2/1 (17) | 0/0 (0) | 0/0 (0) | 2/1 (17) | 0/0 (0) | 0/0 (0) | 4/2 (5) |
| Neutrophil count decreased | 1/1 (17) | 0/0 (0) | 0/0 (0) | 1/1 (17) | 0/0 (0) | 0/0 (0) | 2/2 (5) |
| White blood cell count decreased | 1 1 (17) | 0/0 (0) | 0/0 (0) | 1/1 (17) | 0/0 (0) | 0/0 (0) | 2/2 (5) |
| GENERAL DISORDERS AND ADMINISTRATION SITE CONDITIONS | 0/0 (0) | 0/0 (0) | 0/0 (0) | 0/0 (0) | 2/2 (33) | 1/1 (11) | 3/3 (8) |
| Catheter site bruise | 0/0 (0) | 0/0 (0) | 0/0 (0) | 0/0 (0) | 0/0 (0) | 1/1 (11) | 1/1 (3) |
| Fatigue | 0/0 (0) | 0/0 (0) | 0/0 (0) | 0/0 (0) | 1/1 (17) | 0/0 (0) | 1/1 (3) |
| Vessel puncture site bruise | 0/0 (0) | 0/0 (0) | 0/0 (0) | 0/0 (0) | 1/1 (17) | 0/0 (0) | 1/1 (3) |
| INFECTIONS AND INFESTATIONS | 0/0 (0) | 0/0 (0) | 2/2 (33) | 0/0 (0) | 0/0 (0) | 0/0 (0) | 2/2 (5) |
| Nasopharyngitis | 0/0 (0) | 0/0 (0) | 2/2 (33) | 0/0 (0) | 0/0 (0) | 0/0 (0) | 2/2 (5) |
| MUSCULOSKELETAL AND CONNECTIVE TISSUE DISORDERS | 0/0 (0) | 1/1 (20) | 0/0 (0) | 0/0 (0) | 0/0 (0) | 0/0 (0) | 1/1 (3) |
| Back pain | 0/0 (0) | 1/1 (20) | 0/0 (0) | 0/0 (0) | 0/0 (0) | 0/0 (0) | 1/1 (3) |
| REPRODUCTIVE SYSTEM AND BREAST DISORDERS | 0/0 (0) | 0/0 (0) | 0/0 (0) | 0/0 (0) | 0/0 (0) | 1/1 (11) | 1/1 (3) |
| Metrorrhagia | 0/0 (0) | 0/0 (0) | 0/0 (0) | 0/0 (0) | 0/0 (0) | 1/1 (11) | 1/1 (3) |
| VASCULAR DISORDERS | 0/0 (0) | 0/0 (0) | 0/0 (0) | 0/0 (0) | 1/1 (17) | 0/0 (0) | 1/1 (3) |
| Hot flush | 0/0 (0) | 0/0 (0) | 0/0 (0) | 0/0 (0) | 1/1 (17) | 0/0 (0) | 1/1 (3) |

B.

| System Organ Class Preferred Term | VIT­2763  60 mg QD N=8  E/n (%) | VIT­2763  120 mg QD  N=6  E/n (%) | VIT­2763  60 mg BID N=6 E/n (%) | VIT­2763  120 mg BID N=6 E/n (%) | Pooled placebo QD or BID N=8  E/n (%) | Total N=34 E/n (%) |
| --- | --- | --- | --- | --- | --- | --- |
| ANY ADVERSE EVENTS | 21/8 (100) | 23/5 (83) | 11/5 (83) | 20/6 (100) | 21/5 (63) | 96/29 (85) |
| Nervous system disorders | 7/5 (63) | 6/4 (67) | 1/1 (17) | 2/1 (17) | 8/3 (38) | 24/14 (41) |
| Headache | 4/3 (38) | 3/2 (33) | 1/1 (17) | 2/1 (17) | 3/2 (25) | 13/9 (26) |
| Dizziness | 3/3 (38) | 0/0 (0) | 0/0 (0) | 0/0 (0) | 2/1 (13) | 5/4 (12) |
| Somnolence | 0/0 (0) | 2/2 (33) | 0/0 (0) | 0/0 (0) | 2/2 (25) | 4/4 (12) |
| Disturbance in attention | 0/0 (0) | 1/1 (17) | 0/0 (0) | 0/0 (0) | 0/0 (0) | 1/1 (3) |
| Dizziness postural | 0/0 (0) | 0/0 (0) | 0/0 (0) | 0/0 (0) | 1/1 (13) | 1/1 (3) |
| GENERAL DISORDERS AND ADMINISTRATION SITE CONDITIONS | 2/2 (25) | 2/2 (33) | 3/3 (50) | 9/4 (67) | 3/3 (38) | 19/14 (41) |
| Medical device site reaction* | 0/0 (0) | 1/1 (17) | 1/1 (17) | 4/4 (67) | 1/1 (13) | 7/7 (21) |
| Fatigue | 0/0 (0) | 1/1 (17) | 0/0 (0) | 2/2 (33) | 0/0 (0) | 3/3 (9) |
| Asthenia | 1/1 (13) | 0/0 (0) | 0/0 (0) | 0/0 (0) | 0/0 (0) | 1/1 (3) |
| Catheter site hematoma | 0/0 (0) | 0/0 (0) | 0/0 (0) | 1/1 (17) | 0/0 (0) | 1/1 (3) |
| Catheter site inflammation | 0/0 (0) | 0/0 (0) | 1/1 (17) | 0/0 (0) | 0/0 (0) | 1/1 (3) |
| Catheter site pain | 0/0 (0) | 0/0 (0) | 1/1 (17) | 0/0 (0) | 0/0 (0) | 1/1 (3) |
| Chest pain | 1/1 (13) | 0/0 (0) | 0/0 (0) | 0/0 (0) | 0/0 (0) | 1/1 (3) |
| Chills | 0/0 (0) | 0/0 (0) | 0/0 (0) | 1/1 (17) | 0/0 (0) | 1/1 (3) |
| Malaise | 0/0 (0) | 0/0 (0) | 0/0 (0) | 0/0 (0) | 1/1 (13) | 1/1 (3) |
| Thirst | 0/0 (0) | 0/0 (0) | 0/0 (0) | 0 0 (0) | 1/1 (13) | 1/1 (3) |
| Vessel puncture site pain | 0/0 (0) | 0/0 (0) | 0/0 (0) | 1/1 (17) | 0/0 (0) | 1/1 (3) |
| GASTROINTESTINAL DISORDERS | 4/3 (38) | 5/4 (67) | 2/1 (17) | 2/1 (17) | 3/1 (13) | 16/10 (29) |
| Nausea | 0/0 (0) | 2/2 (33) | 0/0 (0) | 1/1 (17) | 2/1 (13) | 5/4 (12) |
| Abdominal pain | 0/0 (0) | 2/2 (33) | 0/0 (0) | 0/0 (0) | 1/1 (13) | 3/3 (9) |
| Abdominal distension | 0/0 (0) | 0/0 (0) | 1/1 (17) | 1/1 (17) | 0/0 (0) | 2/2 (6) |
| Diarrhea | 1/1 (13) | 1/1 (17) | 0/0 (0) | 0/0 (0) | 0/0 (0) | 2/2 (6) |
| Abdominal discomfort | 1/1 (13) | 0/0 (0) | 0/0 (0) | 0/0 (0) | 0/0 (0) | 1/1 (3) |
| Constipation | 0/0 (0) | 0/0 (0) | 1/1 (17) | 0/0 (0) | 0/0 (0) | 1/1 (3) |
| Frequent bowel movements | 1 1 (13) | 0/0 (0) | 0/0 (0) | 0/0 (0) | 0/0 (0) | 1/1 (3) |
| Gastrointestinal sounds abnormal | 1/1 (13) | 0/0 (0) | 0/0 (0) | 0/0 (0) | 0/0 (0) | 1/1 (3) |
| INVESTIGATIONS | 1/1 (13) | 3/2 (33) | 0/0 (0) | 4/3 (50) | 4/2 (25) | 12/8 (24) |
| White blood cell count decreased | 1/1 (13) | 2/2 (33) | 0/0 (0) | 1/1 (17) | 2/2 (25) | 6/6 (18) |
| Neutrophil count decreased | 0/0 (0) | 1/1 (17) | 0/0 (0) | 2/2 (33) | 2/2 (25) | 5/5 (15) |
| Blood iron decreased | 0/0 (0) | 0/0 (0) | 0/0 (0) | 1/1 (17) | 0/0 (0) | 1/1 (3) |
| MUSCULOSKELETAL AND CONNECTIVE TISSUE DISORDERS | 2/2 (25) | 1/1 (17) | 1/1 (17) | 3/2 (33) | 2/2 (25) | 9/8 (24) |
| Myalgia | 1/1 (13) | 0/0 (0) | 0/0 (0) | 2/1 (17) | 2/2 (25) | 5/4 (12) |
| Back pain | 1/1 (13) | 0/0 (0) | 0/0 (0) | 0/0 (0) | 0/0 (0) | 1/1 (3) |
| Muscle twitching | 0/0 (0) | 0/0 (0) | 1/1 (17) | 0/0 (0) | 0/0 (0) | 1/1 (3) |
| Musculoskeletal pain | 0/0 (0) | 0/0 (0) | 0/0 (0) | 1/1 (17) | 0/0 (0) | 1/1 (3) |
| Pain in extremity | 0/0 (0) | 1/1 (17) | 0/0 (0) | 0/0 (0) | 0/0 (0) | 1/1 (3) |
| REPRODUCTIVE SYSTEM AND BREAST DISORDERS | 1/1 (13) | 1/1 (17) | 1/1 (17) | 0/0 (0) | 1/1 (13) | 4/4 (12) |
| Dysmenorrhea | 1/1 (13) | 1/1 (17) | 0/0 (0) | 0/0 (0) | 0/0 (0) | 2/2 (6) |
| Breast engorgement | 0/0 (0) | 0/0 (0) | 1/1 (17) | 0/0 (0) | 0/0 (0) | 1/1 (3) |
| Metrorrhagia | 0/0 (0) | 0/0 (0) | 0/0 (0) | 0/0 (0) | 1/1 (13) | 1/1 (3) |
| RESPIRATORY, THORACIC AND MEDIASTINAL DISORDERS | 3/2 (25) | 1/1 (17) | 0/0 (0) | 0/0 (0) | 0/0 (0) | 4/3 (9) |
| Epistaxis | 2/2 (25) | 0/0 (0) | 0/0 (0) | 0/0 (0) | 0/0 (0) | 2/2 (6) |
| Oropharyngeal pain | 1/1 (13) | 1/1 (17) | 0/0 (0) | 0/0 (0) | 0/0 (0) | 2/2 (6) |
| INFECTIONS AND INFESTATIONS | 0/0 (0) | 0/0 (0) | 2/2 (33) | 0/0 (0) | 0/0 (0) | 2/2 (6) |
| Nasopharyngitis | 0/0 (0) | 0/0 (0) | 2/2 (33) | 0/0 (0) | 0/0 (0) | 2/2 (6) |
| METABOLISM AND NUTRITION DISORDERS | 0/0 (0) | 2/2 (33) | 0/0 (0) | 0/0 (0) | 0/0 (0) | 2/2 (6) |
| Decreased appetite | 0/0 (0) | 2/2 (33) | 0/0 (0) | 0/0 (0) | 0/0 (0) | 2/2 (6) |
| SKIN AND SUBCUTANEOUS TISSUE DISORDERS | 0/0 (0) | 1/1 (17) | 1/1 (17) | 0/0 (0) | 0/0 (0) | 2/2 (6) |
| Hyperhidrosis | 0/0 (0) | 1/1 (17) | 0/0 (0) | 0/0 (0) | 0/0 (0) | 1/1 (3) |
| Rash macular | 0/0 (0) | 0/0 (0) | 1/1 (17) | 0/0 (0) | 0/0 (0) | 1/1 (3) |
| EYE DISORDERS | 0/0 (0) | 1/1 (17) | 0/0 (0) | 0/0 (0) | 0/0 (0) | 1/1 (3) |
| Eye irritation | 0/0 (0) | 1/1 (17) | 0/0 (0) | 0/0 (0) | 0/0 (0) | 1/1 (3) |
| RENAL AND URINARY DISORDERS | 1/1 (13) | 0/0 (0) | 0/0 (0) | 0/0 (0) | 0/0 (0) | 1/1 (3) |
| Pollakiuria | 1/1 (13) | 0/0 (0) | 0/0 (0) | 0/0 (0) | 0/0 (0) | 1/1 (3) |

Counts are presented in descending order by System Organ Class and Preferred Term (within a System Organ Class) based on the number of participants experiencing the event in the total column

%=Number of events (E) as a percentage of number of participants that experienced an AE in this category (n); N, number of participants exposed per treatment

AE, adverse event; BID, twice daily; QD, once daily

*Relates to medical devices/procedures (such as ECG electrodes reactions, or others) rather than the oral administration of the test drug

**Supplementary Table 5. Summary of all AEs by treatment, relationship and severity for single-ascending dose cohorts (A) and multiple-ascending dose cohorts (B) (safety population).**

A.

| Treatment | ALL AEs | | Unrelated AEs | | Related AEs | |
| --- | --- | --- | --- | --- | --- | --- |
|  | **All severities**  **E/n (%)** | **Mild**  **E/n (%)** | **All severities**  **E/n (%)** | **Mild**  **E/n (%)** | **All severities**  **E/n (%)** | **Mild**  **E/n (%)** |
| VIT-2763 5 mg (N=6) | 3/2 (33) | 3/2 (33) | 0/0 (0) | 0/0 (0) | 3/2 (33) | 3/2 (33) |
| VIT-2763 15 mg (N=5) | 4/3 (60) | 4/3 (60) | 3/3 (60) | 3/3 (60) | 1/1 (20) | 1/1 (20) |
| VIT-2763 60 mg (N=6) | 4/3 (50) | 4/3 (50) | 3/3 (50) | 3/3 (50) | 1/1 (17) | 1/1 (17) |
| VIT-2763 120 mg (N=6) | 5/3 (50) | 5/3 (50) | 2/2 (33) | 2/2 (33) | 3/2 (33) | 3/2 (33) |
| VIT-2763 240 mg (N=6) | 7/4 (67) | 7/4 (67) | 7/4 (67) | 7/4 (67) | 0/0 (0) | 0/0 (0) |
| Pooled placebo (N=9) | 9/5 (56) | 9/5 (56) | 7/5 (56) | 7/5 (56) | 2/2 (22) | 2/2 (22) |
| Total (N=38) | 32/20 (53) | 32/20 (53) | 22/17 (45) | 22/17 (45) | 10/8 (21) | 10/8 (21) |

B.

| Treatment | ALL AEs | | | Unrelated AEs | | | Related AEs | | |
| --- | --- | --- | --- | --- | --- | --- | --- | --- | --- |
|  | **All severities**  **E/n (%)** | **Mild**  **E/n (%)** | **Moderate**  **E/n (%)** | **All severities**  **E/n (%)** | **Mild**  **E/n (%)** | **Moderate**  **E/n (%)** | **All severities**  **E/n (%)** | **Mild**  **E/n (%)** | **Moderate**  **E/n (%)** |
| VIT-2763 60 mg QD (N=8) | 21/8 (100) | 21/8 (100) | 0/0 (0) | 13/6 (75) | 13/6 (75) | 0/0 (0) | 8/3 (38) | 8/3 (38) | 0/0 (0) |
| VIT-2763 120 mg QD (N=6) | 23/5 (83) | 23/5 (83) | 0/0 (0) | 16/4 (67) | 16/4 (67) | 0/0 (0) | 7/4 (67) | 7/4 (67) | 0/0 (0) |
| VIT-2763 60 mg BID (N=6) | 11/5 (83) | 10/5 (83) | 1/1 (17) | 11/6 (100) | 10/5 (83) | 1/1 (17) | 0/0 (0) | 0/0 (0) | 0/0 (0) |
| VIT-2763 120 mg BID (N=6) | 20/6 (100) | 19/6 (100) | 1/1 (17) | 13/6 (100) | 12/5 (83) | 1/1 (17) | 7/3 (50) | 7/3 (50) | 0/0 (0) |
| Pooled placebo QD or BID (N=8) | 21/5 (63) | 20/5 (63) | 1/1 (13) | 15/4 (50) | 15/4 (50) | 0/0 (0) | 6/4 (50) | 5/3 (38) | 1/1 (13) |
| Total (N=34) | 96/29 (85) | 93/29 (85) | 3/3 (9) | 68/26 (76) | 66/24 (71) | 2/2 (6) | 28/14 (41) | 27/13 (38) | 1/1 (3) |

In the SAD phase, no moderate, severe or life-threatening AEs occurred; the related AEs were AEs with the relationship ‘possibly’. None of the related AEs had the relationship ‘probably/likely’ or ‘certainly’. In the MAD phase, no severe or life-threatening AEs occurred; all but 1 of the related AEs were AEs with the relationship ‘possibly’. One of the related AEs had the relationship ‘probably/likely’; none had the relationship of ‘certainly’.

%, number of events (E) as a percentage of the number of participants per treatment (n)

AE, adverse event; BID, twice daily; MAD, multiple-ascending dose; QD, once daily; SAD, single-ascending dose

**Supplementary Figure 1. Study design for the single-ascending dose cohort phase (A) and the multiple-ascending dose cohort phase (B).**


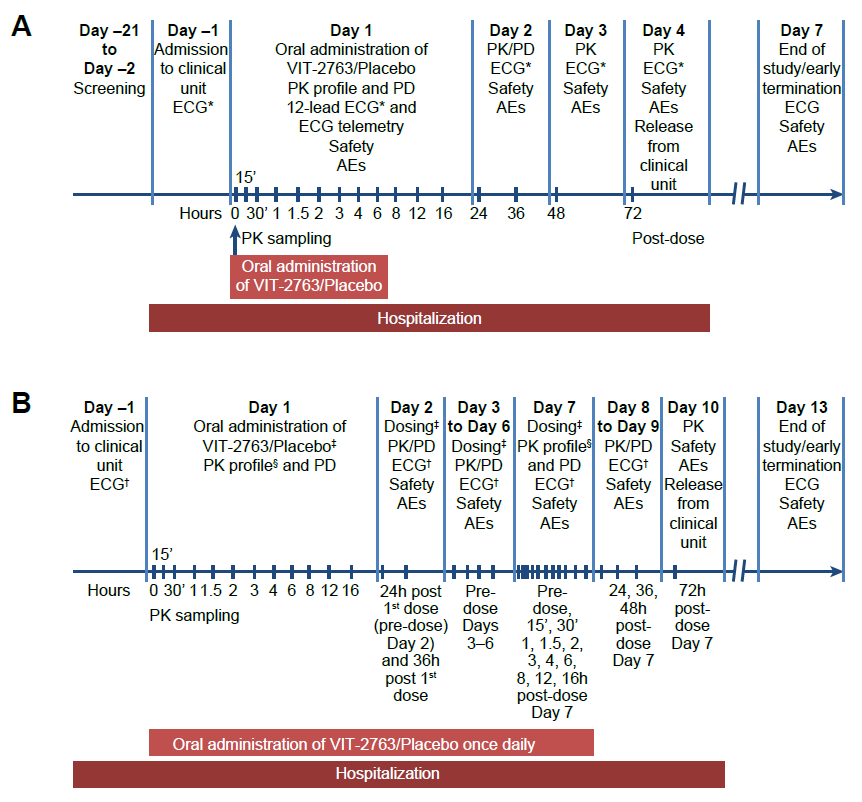


*Triplicate 12-lead ECGs were collected on Day –1, on Day 1 at pre-dose and at 2, 4 and 8 hours post-dose, and on Day 2 at 24 hours post-dose. A single 12-lead ECG was collected on the other days

†Triplicate 12-lead ECGs were collected on Day –1, on Day 1 and 7 before the morning dose and at 2, 4, and 8 hours after the morning dose, and on Day 2 to 6 before the morning dose and at 2 and 4 hours after the morning dose. A single 12-lead ECG was collected on the other days

‡Based on interim PK results of the SAD phase, the dosing regimen of 1 or more dose cohorts of the MAD phase could be adjusted to BID dosing with the evening dose being administered before the evening meal

§Blood samples for PK in cohorts receiving VIT-2763 60 mg QD, VIT-2763 120 mg QD, VIT-2763 60 mg BID, or matching placebo, were collected on Day 1 within 1 hour before the morning dose, at 0.25, 0.5, 1, 1.5, 2, 3, 4, 6, 8, 12, 16 (Day 1), 24 (before the Day 2 morning dose) and 36 hours (Day 2) after the Day 1 morning dose, and then on Day 3 to 7 before the morning dose. Blood samples were collected for a second PK profile on Day 7 in accordance with Day 1 (i.e. within 1 hour before the morning dose and at 0.25, 0.5, 1, 1.5, 2, 3, 4, 6, 8, 12, 16 [Day 7], 24, 36 [Day 8], 48 [Day 9] and 72 hours [Day 10] after the Day 7 morning dose)

For the cohort receiving VIT-2763 120 mg BID or matching placebo, blood samples for PK were collected on Day 1 within 1 hour before the morning dose, at 0.5, 1, 1.5, 2, 3, 4, 6, 8, 12 (before the Day 1 evening dose), 13, 14 (Day 1), 24 (before the Day 2 morning dose) and 36 hours (Day 2) after the Day 1 morning dose, and then on Day 3 to 7 before the morning dose. Blood samples were collected for a second PK profile on Day 7 in accordance with Day 1 (i.e. within 1 hour before the morning dose and at 0.5, 1, 1.5, 2, 3, 4, 6, 8, 12 [before the Day 7 evening dose], 13, 14 [Day 7], 24, 36 [Day 8], 48 [Day 9] and 72 hours [Day 10] after the Day 7 morning dose)

Note: the sampling time points for the VIT-2763 120 mg BID cohort that differed from those for the other cohorts have not been included in the figure

AE, adverse event; BID, twice daily; ECG, electrocardiogram; MAD, multiple-ascending dose; PD, pharmacodynamic; PK, pharmacokinetic; QD, once daily; SAD, single-ascending dose

**Supplementary Figure 2. Disposition of study participants.**


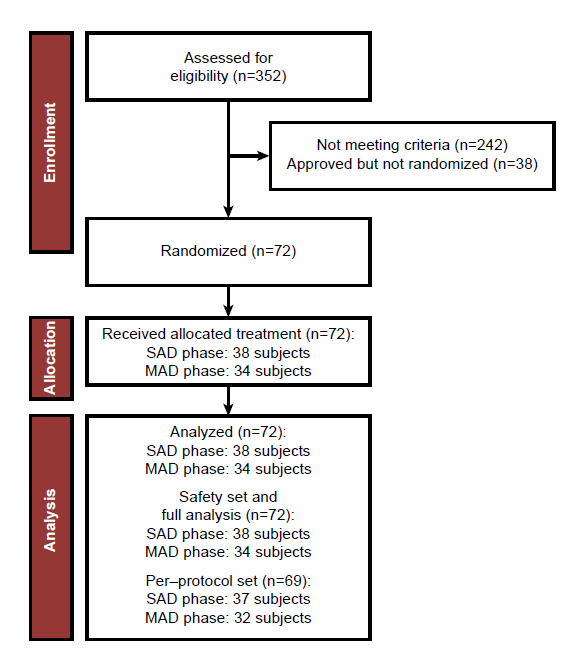


MAD, multiple-ascending dose; SAD, single-ascending dose

**Supplementary Figure 3. Scatter plot of individual AUC_0­last_ versus dose/weight ratio (single- and multiple-ascending dose cohorts; per-protocol population).**

**
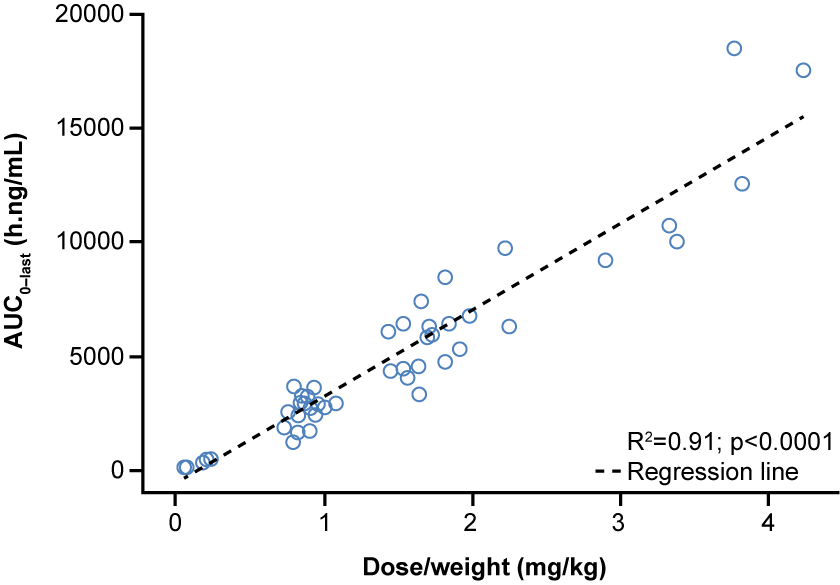
**

AUC_0­last_, area under the concentration–time curve (time 0 to time of last quantifiable concentration)

**Supplementary Figure 4. Scatter plots of individual plasma C_max_ versus dose level/body weight (A), AUC_0-inf_ versus dose/weight (B) and AUC_0­12_ versus dose/weight (C) (single- and multiple-ascending dose cohorts; per-protocol population).**


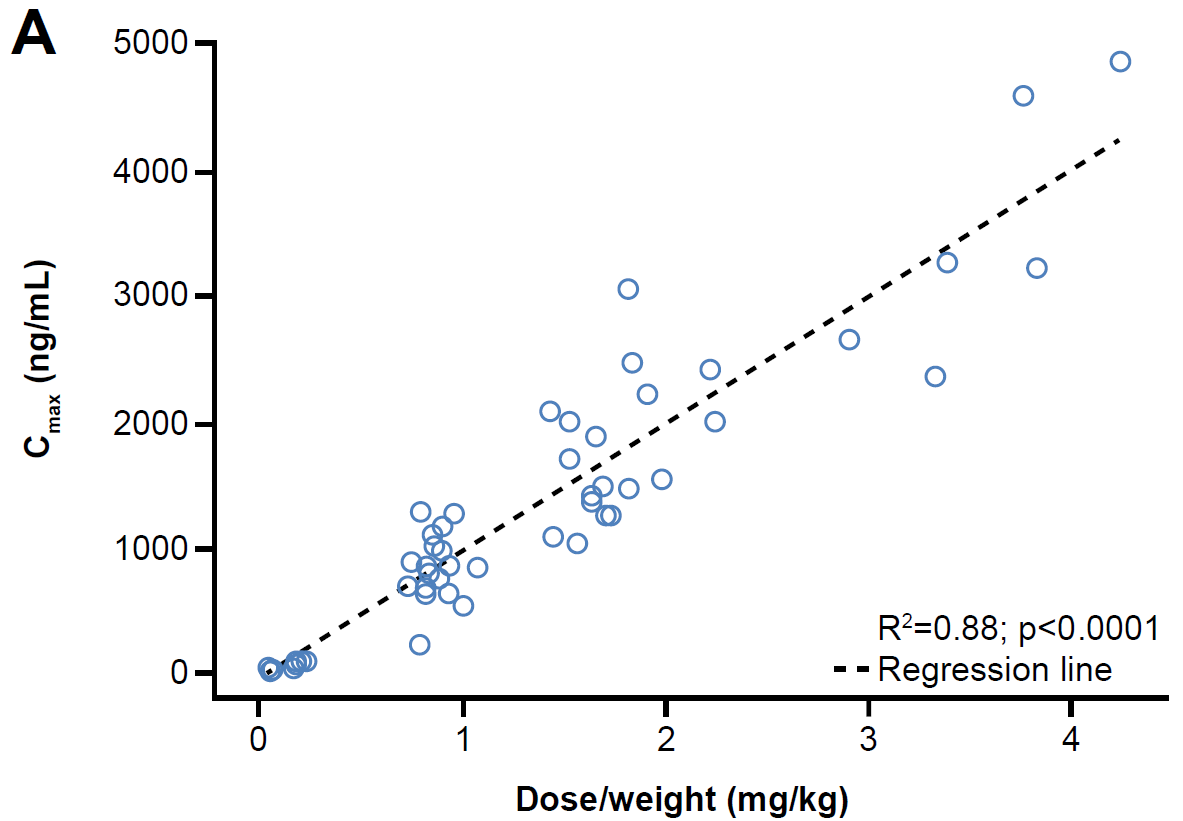


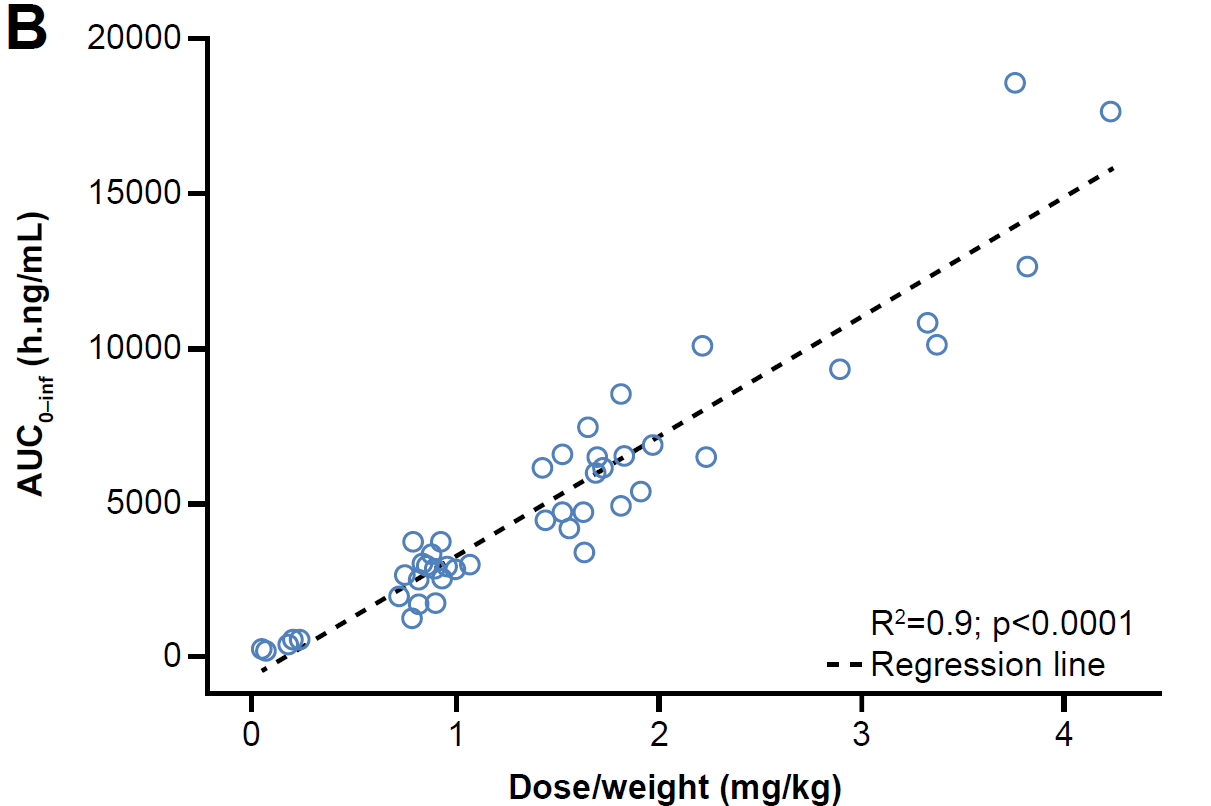


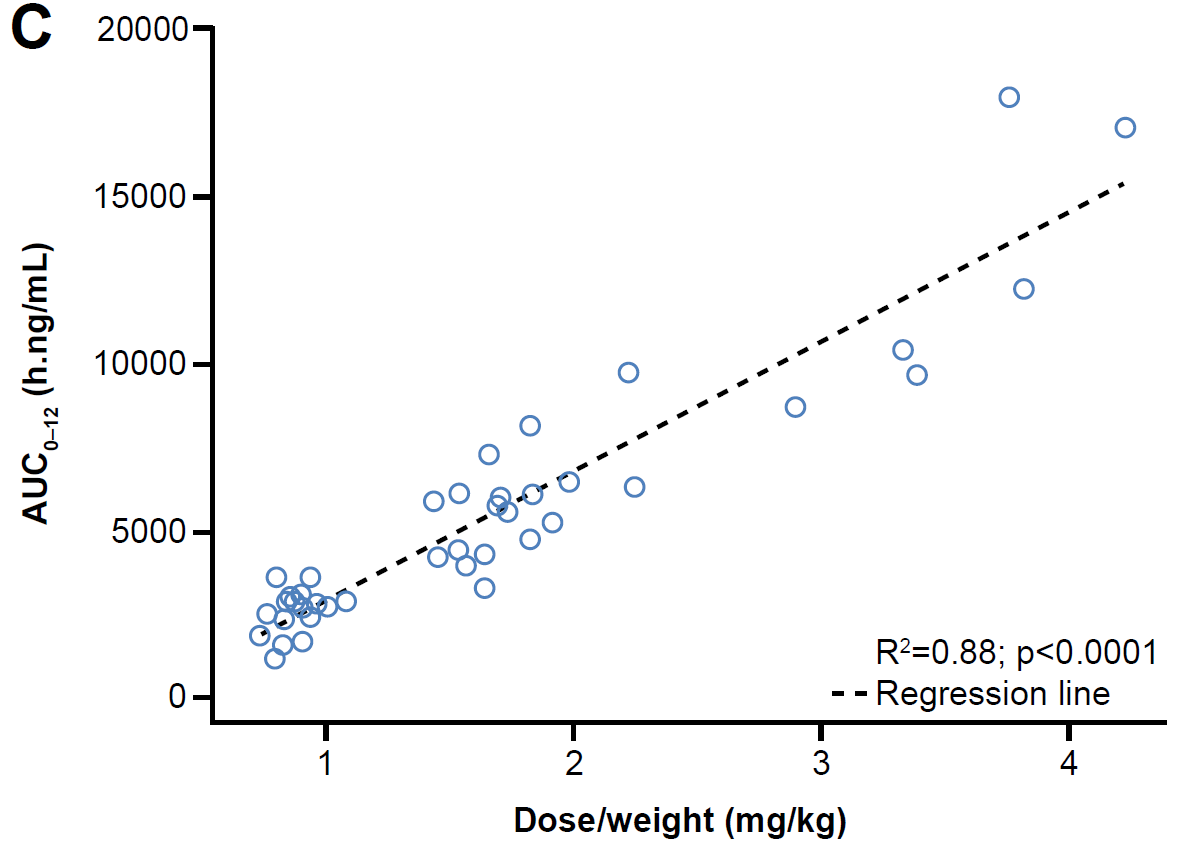


AUC_0-12_, area under the plasma concentration–time curve over the dosing interval (time 0 to 12 hours); AUC_0-inf,_ area under the plasma concentration–time curve over the dosing interval (time 0 to inffinity); C_max_, maximum plasma concentration

**Supplementary Figure 5. Mean serum hepcidin versus time for single-ascending dose cohorts (A) and multiple-ascending dose cohorts on Day 1 (B) and Day 7 (C) (per-protocol population).**


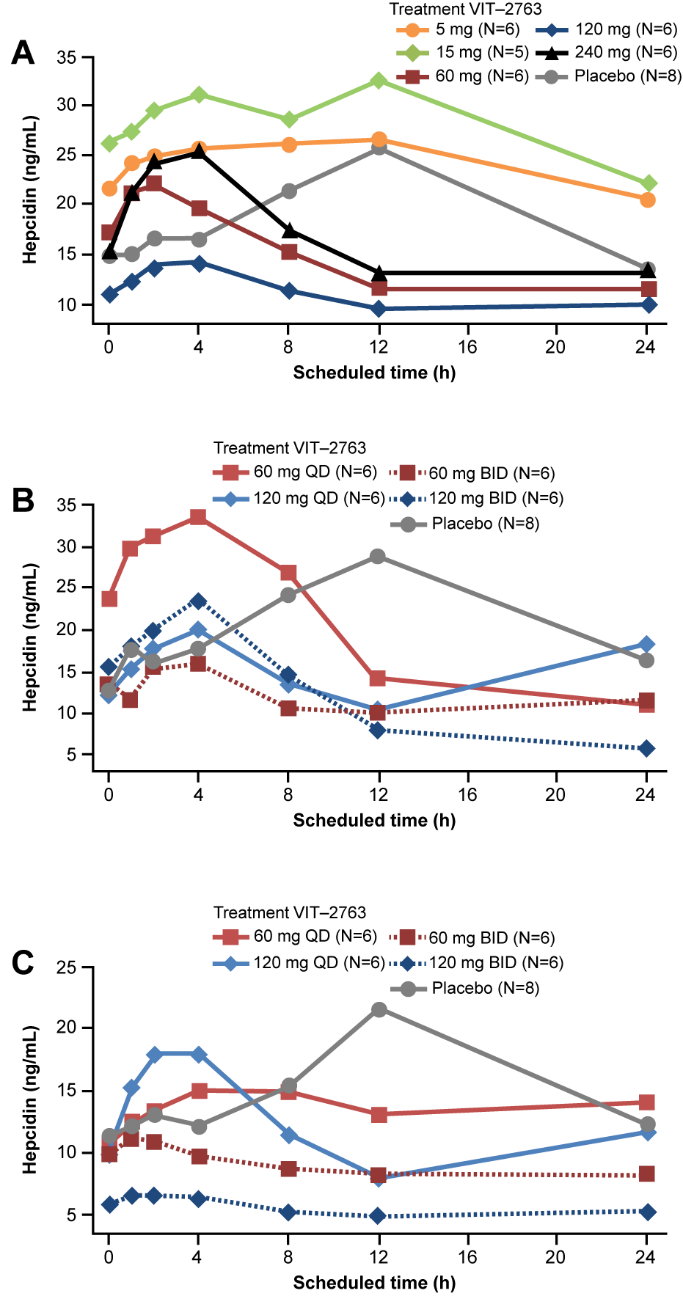


BID, twice daily; QD, once daily

**Supplementary Figure 6. Mean transferrin versus time for multiple-ascending dose cohorts at Day 1 (A) and Day 7 (B) (per-protocol population).**


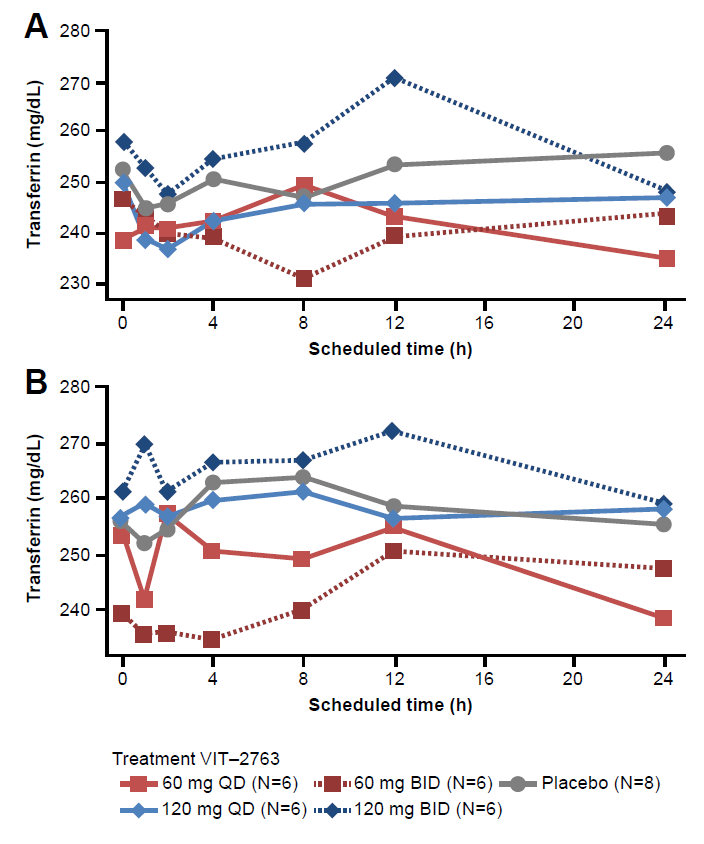


BID, twice daily; QD, once daily

**Supplementary Figure 7. Mean erythropoietin versus time for multiple-ascending dose cohorts at Day 1 (A) and Day 7 (B) (per-protocol population).**


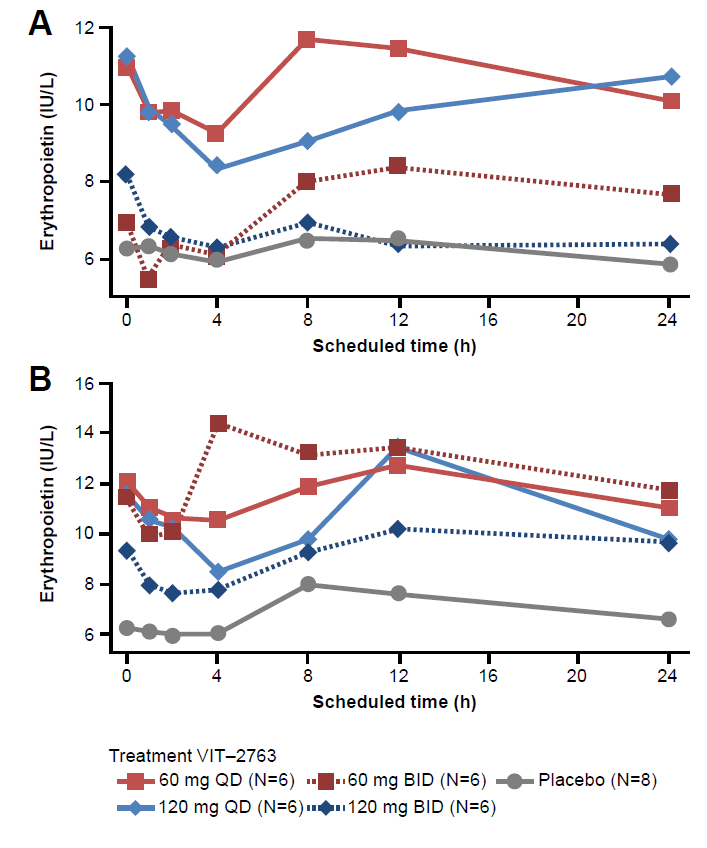


BID, twice daily; QD, once daily

**Supplementary Figure 8. Mean soluble transferrin receptor concentration versus time for multiple-ascending dose cohorts at Day 1 (A) and Day 7 (B) (per-protocol population).**


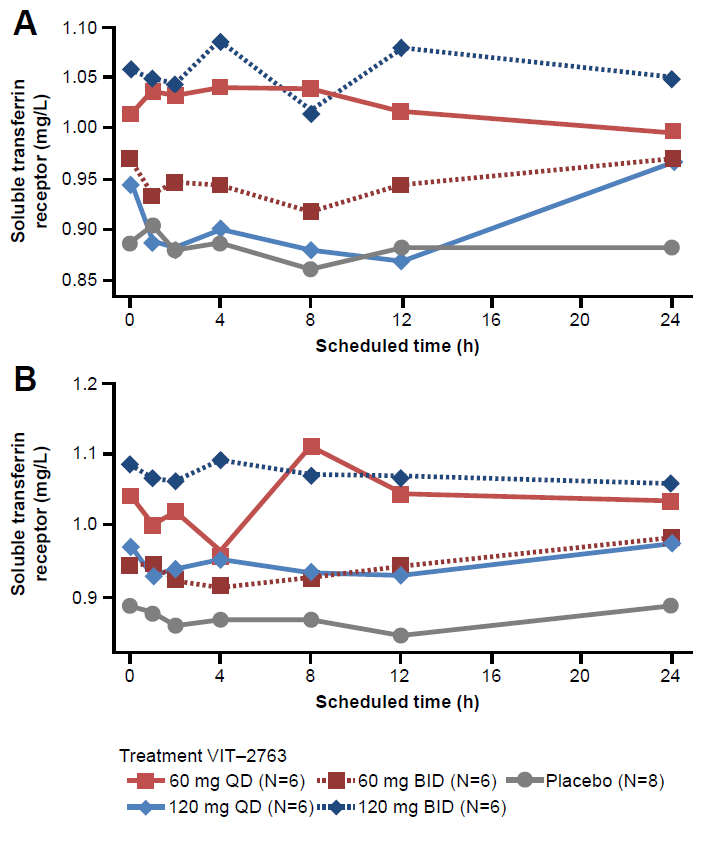


BID, twice daily; QD, once daily
